# A hybrid convolution and attention-based framework with visual explanation for fruit disease identification

**DOI:** 10.1038/s41598-026-42135-5

**Published:** 2026-03-09

**Authors:** Raja Kothandaraman, Saravanan Srinivasan, Sandeep Kumar Mathivanan, Usha Moorthy

**Affiliations:** 1 Department of Computer Science and Engineering, SRM Institute of Science and Technology, Ramapuram, Chennai India; 2 School of Computing Science and Engineering, Galgotias University, Greater Noida, 203201 India; 3 School of Computer Engineering, Manipal Institute of Technology Bengaluru, Manipal Academy of Higher Education, Manipal, Karnataka 576104 India

**Keywords:** Fruit disease, Multi-class fruit classification, DL, Transformer architecture and Grad-CAM, Computational biology and bioinformatics, Engineering, Mathematics and computing

## Abstract

The objective of this study is to create a highly accurate and interpretable deep learning (DL) model for the multi-class classification of fruit using convolutional and transformer architectures. The classification performance can be enhanced by making sure that the used technique is explainable and interpretable. This research data was obtained from Kaggle which contains images of banana, grape, lemon, mango, and strawberry fruit classes. The total data was divided into 70:15:15 for training, validating and testing. To ensure consistent size and quality, all images were pre-processed before use. This study considered four pretrained models namely RegNetY-B3-GE, DarkNet53-SCSE, BEiT, and PVTv2 for performance assessment. We proposed a lightweight hybrid (convolution plus attention-based) CoAT-AgriLite model for fruit disease classification which extracts local lesion features and global context. Transferring training and data augmentation technique was utilized during training for better performance. To ensure interpretability of model decisions, Gradient-weighted Class Activation Mapping (Grad-CAM) which captures the discriminative regions from the input images for model predictions. Among all evaluated models, the proposed model achieved the highest classification accuracy of 99.37% on the testing dataset. Comparative results demonstrated that the proposed model outperformed other pretrained models in terms of precision, recall, and F1-score, confirming its robustness and effectiveness in real-world agricultural classification tasks. The experimental findings validate that the proposed model not only achieves superior classification accuracy but also provides interpretability through Grad-CAM visualizations. This hybrid framework offers a promising solution for intelligent and transparent fruit classification systems, with potential applications in precision agriculture and automated sorting systems.

## Introduction

Fruit plays an important part in human nutrition and economic activity. Fruits are some of the nutrient-dense food items that provide vitamins and minerals along with antioxidants and fibre. They are often recommended as a part of a balanced diet and for preventing diseases. Fruit production is the backbone of the livelihoods of millions – from smallholder farmers to large agribusinesses. It is also a leading source of export revenue in many agricultural economies. Nonetheless, many biotic and abiotic stress factors still affect the ability to produce sufficient volumes and quality of fruit sustainably^1^. A few examples of such microorganisms can be plant pathogenic fungi that cause fruit diseases, bacterial infestation, viral infestation or nutrient deficiency of crops with most being lethal to the health of crops and their marketability. Several fruit crops including bananas, grapes, mangoes, lemons, strawberries, etc. have reported the incidence of anthracnose, powdery mildew, bacterial canker, and black rot diseases^2^. Diseases, usually occur as leaf spots, discoloration, rot, and deformation which lower the beauty and food value of the fruits^3^. If these diseases are not treated, they can quickly spread within the orchard area as well as the outfield area and it can cause heavy yield loss as well. Further, it can also reduce the shelf life and significantly increase the cost of production. This increase in production cost is due to chemical reliance as well as inspection-of-trees labor cost. Consequently, an efficient and precise identification of the diseases of fruit crops will help in minimizing the economic losses, strengthening the food supply system and supporting sustainable agricultural practices^4^. Traditional disease detection approaches are primarily based on manual inspection methods, where trained experts visually inspect the visual symptoms on plant leaves, stems, and fruits. These approaches are helpful; however, they are limited by human emotion and exhaustion, as well as the fact that disease expression can vary from environment to environment and among different crops^5^. Additionally, for larger orchards or in intensive farming systems, manual detection is not scalable and may fail to identify early-stage or asymptomatic infections. To mitigate these drawbacks, the agricultural industry is gradually leaning towards automated and intelligent disease recognition systems based on Machine learning and computer vision^6^.

In particular, the emerging field of DL is a powerful method with a unique ability to discern relevant and non-obvious patterns and visual features from images of fruit with high accuracy. DL is a breakthrough machine learning paradigm that excels at automatically learning explicit hierarchical features from raw image data without extensive user-engineered features^7,8^. They are especially good at addressing the various noisy high dimensions in agricultural images that are taken under different lighting conditions, orientations, and backgrounds. Convolutional Neural Networks (CNNs). Due to their excellent performance in spatial feature extraction, CNNs have become the most widely applicable DL paradigm for visual recognition tasks in agriculture^9,10^. CNNs can learn features associated with disease symptoms on the fruit surface, the local textures, edges and shape of these symptoms^11,12^.

Most recently, transformer-based architectures first popularized for natural language processing have been shown to achieve competitive performance on vision tasks by modelling long-range dependencies and global context in images. Self-attention mechanisms enable a more global evaluation, making them more suitable for capturing spatially distributed features commonly present in diseased fruit images^13^. All the aforementioned successes, however, come with a key challenge traditional DL models are not interpretable, with their infamous black-box nature. The majority of the DL system is a black box; there is very little visibility into how any decision is made. In a high-stakes domain like agriculture, an incorrect classification can lead to economic loss or mismanagement; therefore, model transparency is crucial^14^.

This has resulted in the incorporation of XAI methodologies, such as Grad-CAM and attention heatmaps, which provide visual cues highlighting the image regions that have the most significant influence on the model prediction. These tools will enhance user trust and provide support for a knowledge transfer mechanism by confirming that the model is attending to relevant features of the disease^15^. It is for the above reason that the mix of CNNs, transformers and XAI is such an encouraging step in the right direction for producing strong interpretable real world deployable systems for fruit disease. The systems would help to incorporate drive more transparency as they use algorithms to automated the diagnosis process. This results in creating higher productivity while reducing waste as well as producing healthier fruit. Medium: It enables farmers, agronomists, and supply-chain managers to monitor fruit health^16^.

Despite recent research steps showing that CNN-based and Transformer-based models have been effective for plant disease classification there are still some limitations left. CNN-only approaches tend to have difficulties capturing long-range contextual dependencies while Transformer-based models suffer from costly computations and an absence of inductive biases for fine-grained lesion localization. Furthermore, failure analysis driven by explainability and considerations of practical efficiency are not discussed much. The identified gaps serve as motivation for the present study and for formulating research aim.

The main objectives of this study are given as follows:

i. to develop a lightweight hybrid DL model which should efficiently integrates convolutional inductive biases with attention-based global context modelling in order to obtain the accurate fruit disease classification.
ii. in order to enhance the fine-grained lesion discrimination while concurrently taking long-range spatial dependencies across visually similar fruit classes.
iii. to obtain the good and steady performance under the class imbalance and cross-validation settings along with the computational efficacy appropriate for real-time and the edge deployment.
iv. to integrate explainable AI technique and to specifically Grad-CAM, which is used to provide the appropriate and visually interpretable perceptions into the model predictions. As a result, these objectives directly address the methodological gaps that are identified in the existing models and helps to design and evaluation of the proposed model.

The structure of this study is as follows: Sect. 2 discusses recent state-of-the-art (SOTA) studies related to fruit disease detection, along with their corresponding metric outcomes. Section 3 provides two pieces of information: materials and methods. The material section includes detailed information about the dataset and its image split-up. In contrast, the methods section provides detailed information about four pre-trained CNN models, as well as the proposed hybrid CNN with a transformer model. Section 4 presents the performance metric analysis outcomes of the training, validation, and testing phases for four pretrained and proposed models. Section 5 provides the ablation study outcome of the proposed study, and Sect. 6 discusses the performance metric comparison of the proposed and other SOTA models’ outcomes. Finally, Sect. 7 is the conclusion section, which provides an overview of the proposed study’s limitations and future endeavors.

## Related work

Christian A. Elinisa et al.^17^ used a U-Net DL framework for the segmentation and detection of Fusarium Wilt and Black Sigatoka infected banana leaves and stalks. They trained the model over 18,240 annotated images, as captured by mobile cameras directly on farms under the guidance of experts. U-Net has a good segmentation performance with a Dice score of 96.45% and an IoU of 93.23%. The results reported above ensure the accuracy of the model in identifying disease-affected areas on banana plants.

Fengyun Wu et al.^18^ applied a combination of DL and image processing for banana bunch counting, sterile bud removal, and harvest weight estimation. Here, we applied Deeplab V3 + among classic algorithms for bunch segmentation and counting on bunches at various growth stages, achieving an overall counting accuracy of 93.2%. Development of a software tool for the estimation of the weight of fruits during harvest.

Sahana Shetty et al.^19^ trained an HCA-YOLOv8 Model for the Detection of Banana Plant Diseases using Channel Attention and Improved Deformable Convolution in the Network Backbone. Performance was further improved by performing hyper-parameter tuning using the enhanced Chimera optimization algorithm. All experiments were conducted in Python. The model performed well, achieving 98.12% accuracy on the BananaLSD dataset, 99% on the banana leaf dataset, and 98.5% on the PSFD-Musa dataset.

Ruiheng Li et al.^20^ continue to develop an advanced DL approach for grape disease detection, based on multiple datasets and parallel heterogeneous activation functions, to increase accuracy and robustness. The model outperformed established models, such as YOLOv3, YOLOv5, DETR, TinySegformer, and Tranvolution-GAN, achieving 91% (mAP) in accuracy, 93% and 90% (mAP) for precision and recall, respectively, and reaching a final mAP of 91% at a real-time processing speed of 56 FPS.

Yizong Wang et al.^21^ developed a model to simultaneously identify diseases in grape leaves and fruit. Disease images, of leaves, fruits, and both, are harvested and sent forth to the detection system, in which the YOLOv5s method can auto-detect diseased areas and annotate diseased areas. It was trained on 2,870 images across seven disease classes, with 711 images serving as the test set for evaluation. The validation result indicates that the average potency is 95.6% for all types of diseases, allowing for effective and precise monitoring of diseased plants in the grape production process.

Yifan Liu et al.^22^ present FTR-YOLO, a lightweight, high-accuracy detection model based on a VoVNet backbone that employs ghost convolutions, along with a learnable downsampling layer. To improve detection accuracy while maintaining reconstructive efficiency, squeeze-and-excitation modules combined with residual connections are embedded into the OSA block to form an enhanced OSA block. Finally, a redesigned dual-stream PAN + FPN framework, combined with the Real-time Transformer, is proposed by substituting 3D position embeddings with 2D ones and adopting a Transformer Encoder to enhance the capacity for small object detection and real-time performance at the neck. A new and improved Task-Aligned Predictor in a Decoupled Head design offers a significant trade-off between performance and speed. FTR-YOLO achieves a mAP of 90.67% in the experiments and it proves to be competitive once again.

A K M Fazlul Kobir Siam et al.^23^ developed a comprehensive dataset on lemon leaf disease to promote agricultural research and enhance the management of the disease. A total of 1,354 original images were collected from Charpolisha under Jamalpur District, Bangladesh, from July to September 2024, with the help of field experts. These images were later augmented to generate 9,000 images using flipping, rotation, zooming, shifting, noise, shearing, and brightening. Images were resized to 800 × 800 pixels and divided into nine classes related to different diseases and healthy leaves. The model achieved an accuracy score of 98.56% with an augmented dataset and 96.19% without an augmented dataset, highlighting the importance of an augmented dataset in effectively classifying the disease.

Nouman Butt et al.^24^ proposed a DL-based automatic citrus disease classifier by optimal feature selection. Using data augmentation and transfer learning, refined models based on DenseNet-201 and AlexNet yielded a high accuracy score of 99.2% with citrus leaf images. It smacks the chocolates of earlier approaches and provides an efficient and scalable framework for sustainable disease detection in citrus plantations.

Archna Goyal et al.^25^ compared EfficientNetB0, ResNet-50, DenseNet-121, and Inception-V3 on 759 images belonging to nine classes to classify citrus diseases. Of these, DenseNet-121 and InceptionV3 both gave the best results, yielding 99.12% test accuracy, along with high F1 Scores. On the contrary, ResNet50 and EfficientNetB0 have lower accuracies of 84.58% and 80.18% respectively.

Yohannes Agegnehu Bezabh et al.^26^ presented an ensemble CNN model that combines GoogLeNet and VGG16 models for image classification. Its transparent workflow encompassed the data collection, preprocessing, segmentation, and feature extraction steps. With accuracies reaching up to 99.87% (train), 99.72% (validation), and 99.21% (test), the performance of the fused models confirms the viability of using ensemble methods.

Muhammad Attique Khan et al.^27^ proposed a method for plant disease detection that consists of segmentation, feature extraction and fusion, feature selection, and classification. The infected areas are segmented based on a single Artificial Quintessence Evolutionary algorithm with adaptive quartile deviation and fused as a weighted cross-correlation coefficient. They select key features by employing a new entropy and rank-based correlation framework and finally classify them using a multi-class support vector machine. Extensive evaluation on the PlantVillage dataset yielded promising results, with an average segmentation accuracy of 93.74% and classification accuracy of 97.7%, indicating the efficiency of the steps in the proposed pipeline.

Yi-Chen Chen et al.^28^ propose a multi-scale and multi-pooling convolutional neural network (MSMP-CNN) for detecting mango leaf disease from real images. Initially, transfer learning along with fine-tuning was implemented for the model. The MSMP-CNN alone achieved 95% accuracy, which increased to 98.5% after transfer learning and fine-tuning. To further evaluate the effectiveness of these enhancements, t-SNE visualizations were created to compare classification performance under both conditions.

**Table 1:** Systematic analysis of representative related works and their key limitations in fruit disease detection.

Authors	Models	Primary task	Key observations
^17^	U-Net	Segmentation	High segmentation accuracy, but computationally intensive and not designed for lightweight classification or real-time deployment
^18^	DeepLabV3+	Segmentation	Effective for object-level tasks; however, limited effectiveness for fine-grained disease classification
^19^	HCA-YOLOv8	Detection	Strong detection accuracy, but model complexity and hyperparameter sensitivity reduce deployment efficiency
^20^	Multi-activation DL	Detection	Improved robustness across datasets; however, detection-focused design limits class-level disease discrimination
^21^	YOLOv5s	Detection	Efficient and fast, but limited capacity to capture subtle texture-based disease symptoms
^22^	FTR-YOLO	Detection	Lightweight and real-time capable, yet primarily optimized for object detection rather than disease classification
^23^	Custom CNN	Classification	High accuracy on controlled datasets, but lacks explicit global context modeling and explainability
^24^	DenseNet-201 + AlexNet	Classification	Ensemble improves accuracy but significantly increases parameter count and inference cost
^25^	DenseNet-121/InceptionV3	Classification	Strong feature learning, but purely convolutional architecture limits long-range dependency modeling
^26^	GoogLeNet + VGG16	Classification	High performance through fusion, but computationally heavy and lacks interpretability mechanisms
^27^	Feature-fusion + SVM	Classification	Effective pipeline, yet depends on handcrafted feature engineering stages and lacks end-to-end learning transparency
^28^	MSMP-CNN	Classification	Multi-scale pooling enhances robustness; however, absence of attention limits global contextual understanding

The systematic analysis shown in Table 1 demonstrates that the existing models mainly highlight either localized feature extraction or object-level detection, though inadequately addressing long-range contextual dependencies, computational efficiency, and model interpretability in a unified model. However, the CNN-based methods show better texture insight but struggle with global disease patterns, while detection-oriented models prioritize the bounding accuracy over fine-grained classification. Additionally, most of the studies lag in clear explainability technique, preventive transparency and real-world implementation. In addition, these limitations directly encourage the proposed model, which integrates the convolutional inductive biases with attention-based global modelling and explainable AI to address these challenges efficiently.

### Proposed study vs. SOTA models

Recent advances in detection of plant diseases have applied various sophisticated DL techniques. Each method employs distinctive designs and structural choices that influence how it works. Models like U-Net, DeepLab V3+, and versions of YOLO focus on separating and detecting objects using networks of neurons. These structures can pick out the minutiae but may fail to see how the bits and pieces relate to each other and the whole, which is essential given that symptoms can be diffuse or subtle. Another techniques such as HCA-YOLOv8, FTR-YOLO, and advanced groups of CNNs (GoogLeNet + VGG16) introduce channel attention and flexible connection adjusts or lighter frameworks to improve problem and speed detection. Nonetheless, most still rely on convolutional layers with limited effectiveness for adapting to the varying size, shape, and orientation of symptoms and modelling long-range relationships. Although BEIT and PVTv2 are transformer-based models using self-attention to create a global context and capturing interactions between features, such data-hungry and resource-intensive model may not focus on local patterns that are generally required for early/small-scale symptom catching. On top of that, earlier works often do not target a high classification accuracy and interpretability. Many function as inscrutable black boxes, greatly curtailing the capacity to comprehend or validate the rationale for predictions, which is critical in deploying agricultural technologies responsibly and ensuring farmer trust. This approach forgoes transparency for accuracy, yet agriculture needs both for long sustainability. The proposed CoAT-AgriLite hybrid model is explicitly designed to address these architectural and practical shortcomings:

- Hybrid Feature Learning: By integrating a convolutional stem with transformer-based attention blocks, the model captures both the local detail (essential for subtle or early disease symptoms) and the global context (vital for complex or distributed patterns), bridging the gap between pure CNNs and pure transformers.
- Cross-Scale and Orientation-Aware Attention: The use of convolutional-oriented attention and cross-scale feature fusion enables robust recognition of symptoms at varying sizes, shapes, and orientations something many SOTA models are not optimized for.
- Edge and Field Readiness: The architecture is lightweight and efficient, supporting real-time operation and deployment on resource-limited devices, which is often a limitation in transformer-dominated or ensemble approaches.
- Transparency: Explainable AI modules, such as Grad-CAM, are incorporated to provide visual interpretation of model predictions, making the system not only accurate but also trustworthy and practical for agricultural stakeholders.

##  Materials and methods

**Fig. 1:**
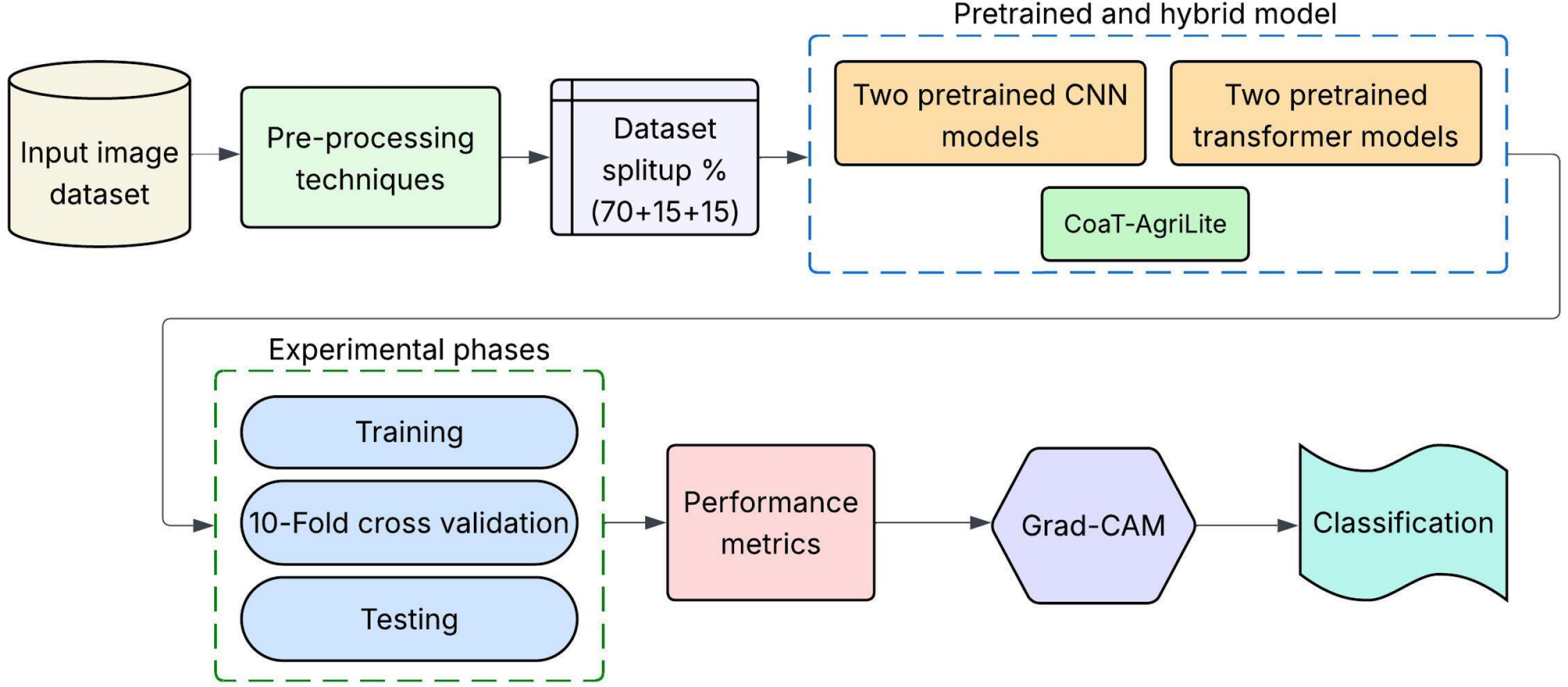
Overall working structure of the proposed study.

This section describes essential aspects utilized within the creation and validation of the classification framework. The approach consists of four main stages comprising dataset preparation with training and test sets, image pre-processing, benchmarking on pre-trained models, and a new hybrid DL architecture proposal. The dataset is arranged in class wise manner and well-balanced which helps in a fair distribution of classes for model evaluation. Techniques that manipulate the input image data are termed pre-processing. They are meant to change input image size, to increase the generalization power of the features learned, and to add augmented samples. A set of pre-trained models is utilized to fine-tune the classification task for benchmark results. These models have altogether different architectural embeddings, which will act as the comparison backdrop for measuring the proposed approach. At the heart of this design is a composite model that combines convolutional layers with transformer-based attention mechanisms, which aims to capture local texture patterns and global contextual relationships. The general approach seeks to achieve three properties, accuracy, scalability, and interpretability, which are consistent with the needs of intelligent agriculture. Figure 1 represents the overview of the proposed study.

### Materials

**Fig. 2:**
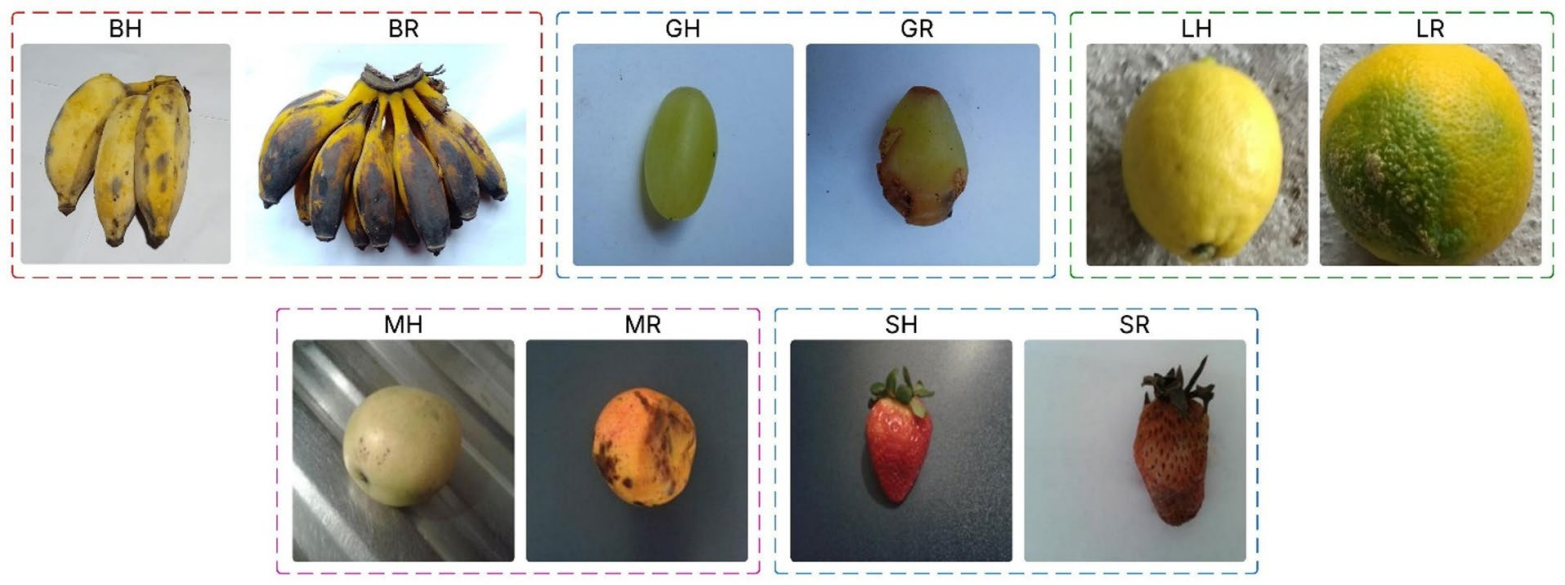
Sample healthy and rotten fruit images from the dataset.

The dataset used in the current study comprises 22,457 images from ten different categories, representing healthy and rotten states of five fruits: bananas, grapes, lemons, mangos, and strawberries (as shown in Table 2). To structure training and evaluate the performance of the learned models effectively, we split the training session into three parts: 70% of sequences for training, 15% for validation, and 15% for testing. This stratified split was performed consistently across all classes to preserve the proportion of classes in the learning model and avoid class imbalance in the modelling^29^. The data consists of 2,000 healthy banana images and 2,800 rotten banana images, 200 healthy grape images and 200 rotten grape images, 5,000 healthy lemon images and 5,000 rotten lemon images, 1,813 healthy mango images and 2,247 rotten mango images, 1,602 healthy strawberry images and 1,595 rotten strawberry images. The carefully crafted and well-distributed dataset enables the condition of fruit samples to be widely represented, which is crucial for obtaining robust classification performance for deployment in real-life use. The sample images of healthy and rotten fruits from the dataset are shown in Fig. 2.

**Table 2:** Dataset summary with fruit disease classes.

Class	Train (70%)	Valid (15%)	Test (15%)	Total
Banana Healthy (BH)	1400	300	300	2000
Banana Rotten (BR)	1960	420	420	2800
Grape Healthy (GH)	140	30	30	200
Grape Rotten (GR)	140	30	30	200
Lemon Healthy (LH)	3500	750	750	5000
Lemon Rotten (LR)	3500	750	750	5000
Mango Healthy (MH)	1269	272	272	1813
Mango Rotten (MR)	1573	337	337	2247
Strawberry Healthy (SH)	1122	240	240	1602
Strawberry Rotten (SR)	1117	239	239	1595

#### Pre-processing techniques

To ensure uniformity across the dataset and prepare the input images for DL architectures, a sequence of preprocessing techniques was applied^30^. The first step involved image resizing with aspect-ratio preservation, where all images were resized to a fixed resolution of 224 × 224 pixels using bilinear interpolation. If the original aspect ratio differed from 1:1, padding was applied using the dataset’s mean pixel value to maintain visual consistency without distortion. Next, a color space conversion was performed, converting all images from BGR to RGB, as per the input standard of most DL models. This was followed by min-max normalization, where pixel values $p\in\left[\mathrm{0,255}\right]$ were scaled to range [0, 1] using the following equation,

1 $${p}_{norm}=\frac{p}{255}$$

To enable transfer learning using pretrained models, channel-wise normalization was performed using the ImageNet statistics. The normalized pixel value ${x}_{norm}$ for each channel was computed as,

2 $${x}_{norm}=\frac{x-\mu}{\sigma}$$

Where µ = [0.485, 0.456, 0.406] and σ = [0.229, 0.224, 0.225] represent the mean and standard deviation for RGB channels, respectively, to address variations in illumination and improve contrast, Contrast-Limited Adaptive Histogram Equalization (CLAHE) was applied to the luminance channel. CLAHE enhances local contrast while preventing noise amplification using a clip limit of 2.0 and a tile grid size of (8, 8). To further improve generalization and mitigate overfitting, several data augmentation techniques were applied during training. These included: (1) Random rotation up to ± 30°, (2) Horizontal and vertical flipping with 50% probability, (3) Scaling and cropping, where the image was scaled by a random factor $s\in[0.8,1.2]$ and cropped back to 224 × 224 pixels, (4) Translation up to ± 10% in both x and y directions, (5) Color jitter by adjusting brightness, contrast, saturation, and hue, and (6) Gaussian noise injection, where zero-mean Gaussian noise $\mathcal{N}(0,{\sigma}^{2})$, with $\sigma\in\left[0,0.05\right]$ was added to simulate sensor noise. Subsequently, the augmented images became PyTorch tensors with shape (3, 224, 224). Moreover, images were checked for numerical stability by removing all infinite values. Through a structured preprocessing pipeline, we standardize the input such that the signal is amplified and variability increases across the training samples.

**Pseudocode:**
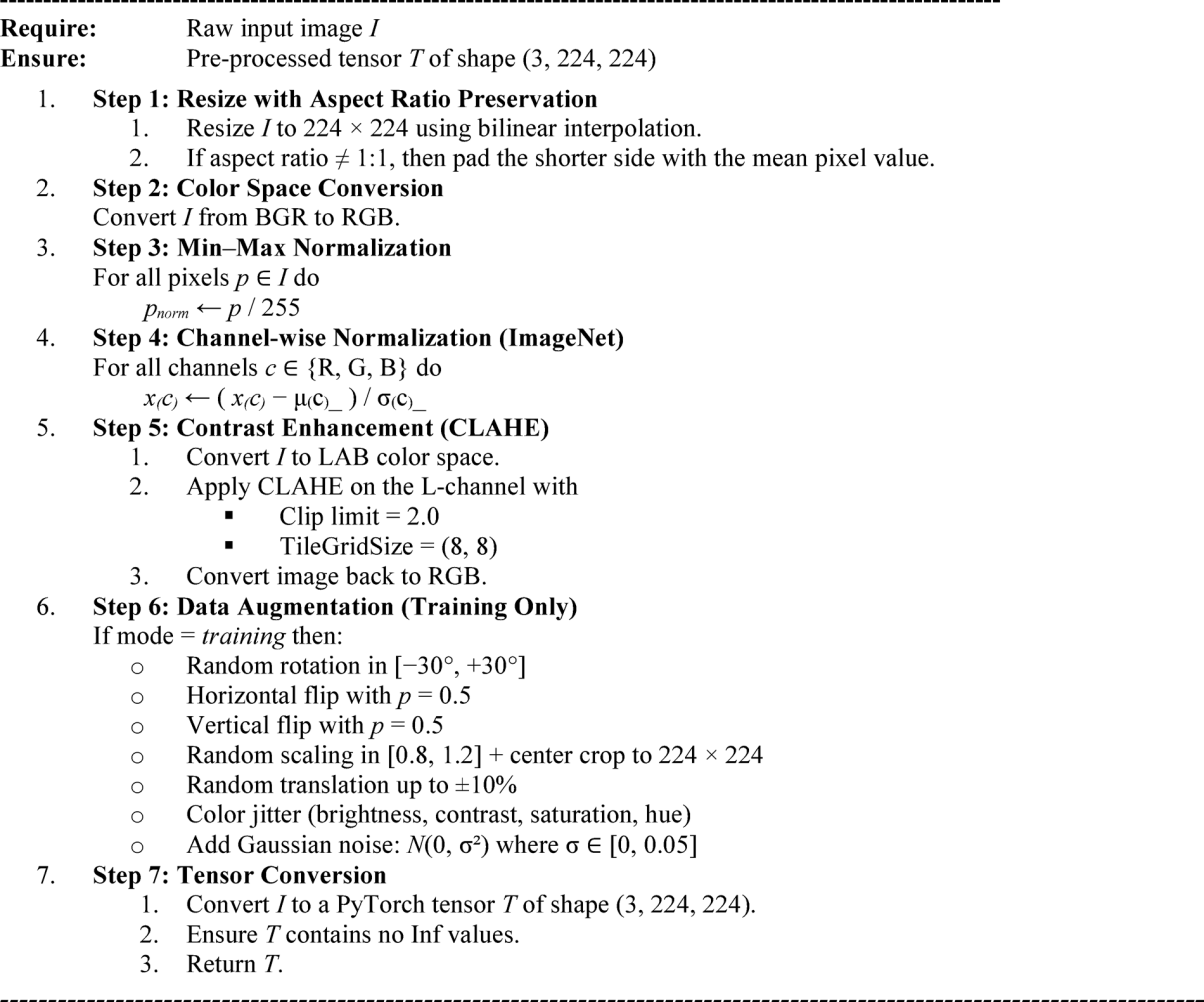
Preprocessing technique.

The pseudocode for a DL-based fruit image pre-processing routine is shown below. This code is a pseudocode example for fruit classification using DL. This process begins with input standardization, including resizing and color space conversion, followed by normalization in preparation for a pre-trained model. CLAHE is an enhanced local contrast method, which is only used in the desired ROI. The pipeline includes a variety of augmentations, such as geometric transformations, intensity and noise simulation, incorporated during training to increase robustness. Finally, you output a clean normalized tensor ready for model ingestion.

### Methods

To enhance generalization ability, four pre-trained CNN models are utilized in this work as strong baselines with a reliable feature extraction capacity across various visual domains. Additionally, a hybrid model CoAT-AgriLite was introduced by combining convolutional attention with the transformer-based structure to preserve both local and global features better. As a result, the Transformer captures long-range dependencies and contextual information, while the CNN layers are specially designed to learn fine-grained spatial patterns. It is believed that the use of this hierarchical recognition scheme can increase classification accuracy and generalization performance in application to fruit disease recognition.

#### RegNetY-B3-GE

We present RegNetY-B3-GE, a lightweight model that demonstrates respectable performance on various visual benchmarks. Consequently, RegNetY-B3-GE was used as one of the benchmark models for fruit disease detection^31^. RegNet derives from a network design space built on a design principle for network design. By imposing strong design constraints on a network, its design space can be simplified and made more effective. Such a design creates uniform networks which are easy to scale-up. Summarily refers to in short. The B3 architecture comprises ~ 12.6 million parameters across around 39 layers, spanning eight stages. It uses the bottleneck blocks with group convolutions and a stride of 2 to achieve hierarchical feature extraction. It takes an input image of size 224 × 224 and provides deep feature embedding, which is then fine-tuned by a custom classification head for fruit disease detection. Its sensitivity to detailed spatial characteristics makes it a strong baseline for evaluating the performance of the proposed hybrid model.

#### DarkNet53-SCSE

DarkNet53-SCSE is used as a reference model in this work due to the good trade-off between the representational capacity and computational cost^32^ for fruit disease detection. In contrast to the classic DarkNet53 backbone widely adopted by the YOLOv3 object detection framework, our attention-augmented backbone, named SCSE, is endowed with the capability of refining attention in both spatial and channel-wise dimensions. The base model, DarkNet53, consists of 53 convolutional layers and is constructed of residual blocks, totalling approximately 41 million parameters. It employs consecutive 3 × 3 and 1 × 1 convolutions, accompanied by shortcut connections, which help maintain the gradient flow and learn features more deeply. The SCSE attention mechanism enables the network to adaptively rescale features, thereby enhancing its ability to focus on useful fruit regions with disease. At an input resolution of 224 × 224 pixels, the DarkNet53-SCSE generates deep embeddings that enable high-quality learning, followed by a task-specific fully connected head for classification. Its dual-attention structure also fits well with the detection of subtle disease cues among complex fruit images.

####  BEiT

BEiT (Bidirectional Encoder representation from Image Transformers) is employed in this work to examine the applicability of transformer-based architectures for fruit disease classification^9^. As a vision transformer (ViT) model, BEiT is pre-trained through masked image modelling, enabling it to capture high-level semantic information without requiring large-scale labeled data. The model tiles patches from each input image and linearly embeds them, before passing them through multiple self-attention layers. The BEiT variant we utilized in the study consists of approximately 86 million parameters, with a base configuration of 12 transformer blocks, a 768-dimensional hidden size, and 12 self-attention heads. It takes as input images downsized to 224 × 224 pixels: these images are tiled into 196 patches (including the class token). Due to its ability to capture global context, BEiT is well-suited for detecting subtle and spatially spread disease symptoms across fruit surfaces. Its incorporation also serves to build a strong transformer-only baseline for comparison with CNN and hybrid models.

#### PVTv2

We utilize Pyramid Vision Transformer v2 (PVTv2) as a robust transformer-based baseline for fruit disease detection, which combines the discriminative capacity of transformers with the multi-scale processing capability of convolutional networks^10^. An additional hierarchical architecture with increasingly smaller spatial resolutions is introduced in PVTv2, which can more effectively free responses to depict fine-scale details and global information as well. Our model extends the original PVT by adding linear attention, SRA, and improved feedforward blocks to enhance both the representation capacity and computational efficiency for large-scale perception tasks. The PVTv2-B2 variant used in our study has a total number of ~ 25 M parameters, which are structured in four stages of depth-to-width increasing (64, 128, 320, 512). There are multiple transformer blocks with multi-head self-attention (MHSA) and patch embeddings in each stage. Throughout the remainder of this study, lowercase and uppercase letters will be used to represent the input images and their features, respectively. The input images are resized to 224 × 224 pixels, and localized features across different scales are extracted, aggregated, and provided as input to the classification head. As PVTv2 is capable of coordinating long-range dependencies without sacrificing efficiency, it applies to complex image analysis tasks, such as disease ROI detection in fruit images.

#### Proposed hybrid model

**Fig. 3:**
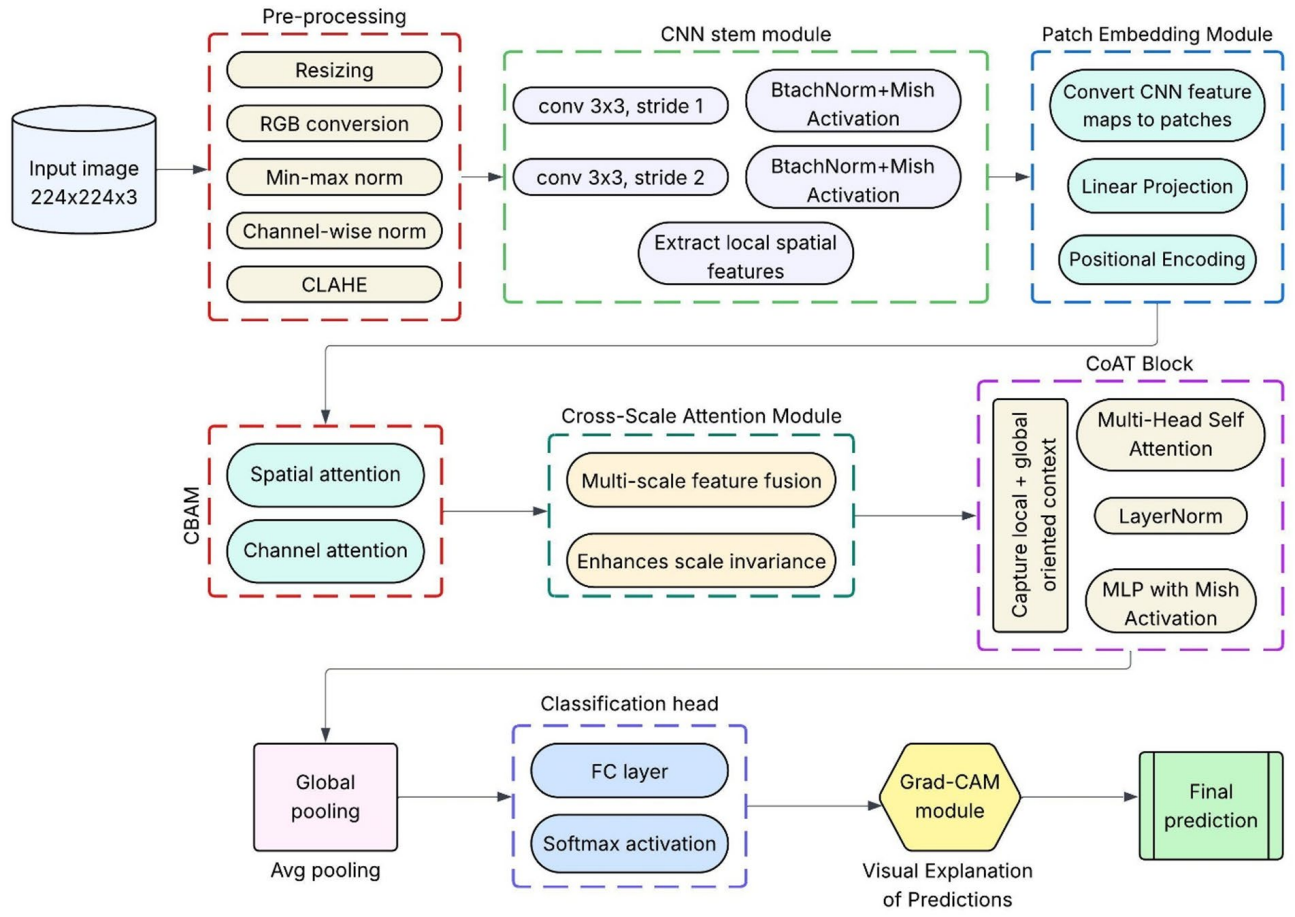
Proposed study workflow.

The present traditional hybrid CNN–Transformer models which combines the feature extractors in a sequential or late-fusion method; however, the proposed model is used to adopts a feature-level technique which is used to embeds the convolutional inductive biases directly within attention-based token processing. This specific design which directly enables the orientation-aware and cross-scale feature interactions whereas to maintain a compact parameter footprint, furthermore, it addressing the dual task of accurate disease classification and computational efficiency. In this study, we propose CoAT-AgriLite, a task-specific hybrid DL model for fruit disease detection, designed to effectively capture both fine-grained local features and broader contextual patterns commonly found in agricultural disease manifestations, as shown in Fig. 3. The proposed model is termed CoAT-AgriLite, where CoAT denotes the integration of Convolutional operations with Attention-based Token processing, and AgriLite reflects the model’s lightweight design tailored for agricultural disease classification tasks. The architecture combines convolutional layers for efficient local feature extraction with attention mechanisms to capture global contextual dependencies, while maintaining a compact parameter footprint. This design choice enables CoAT-AgriLite to achieve high classification accuracy while remaining computationally efficient and suitable for practical deployment scenarios. The motivation behind CoAT-AgriLite stems from the inherent complexity and variability of fruit disease symptoms, which may appear as small-scale localized lesions or large-scale patterns, often occurring at varying orientations and scales. Traditional CNN architectures are highly effective in learning translation-invariant local features but often struggle to capture long-range dependencies without significant depth and computational overhead. Conversely, Vision Transformers (ViTs) offer powerful global context modelling but tend to lack inductive biases, such as locality and hierarchy, leading to overfitting in small or medium-sized datasets and requiring extensive data and computational resources. To address these limitations, CoAT-AgriLite leverages the CoAT by integrating convolutional design priors into the transformer’s attention, resulting in a synergistic setup that borrows the best from both CNN and transformer. The architecture begins with a lightweight CNN stem to encode detailed spatial textures and edge features, which are crucial for recognizing early signs of fruit disease on fruit surfaces. We employ a sequence of CoAT attention blocks, which utilize relative position encoding and convolutional orientation-aware attention to robustly capture geometric variations, such as rotated or deformed symptoms.

**Table 3:** Mapping of identified challenges to proposed design solutions.

Identified challenge	Limitations in existing models	Proposed solution in CoAT-AgriLite
Limited global context	CNN-only models	CoAT-based cross-scale attention
Weak lesion localization	Transformer-only models	CNN inductive priors + mid-fusion
High computational cost	Heavy ensembles	Lightweight hybrid design
Lack of interpretability	Black-box DL models	Integrated Grad-CAM

In order to clearly connect the addressed challenges in the traditional fruit disease detection techniques with the proposed architectural technique, here the Table 3 demonstrates the important limitations and the appropriate solutions which are incorporated in the CoAT-AgriLite framework. As a result, this mapping exhibits how each component of the proposed model is directly inspired by a specific methodological gap.

**Fig. 4:**
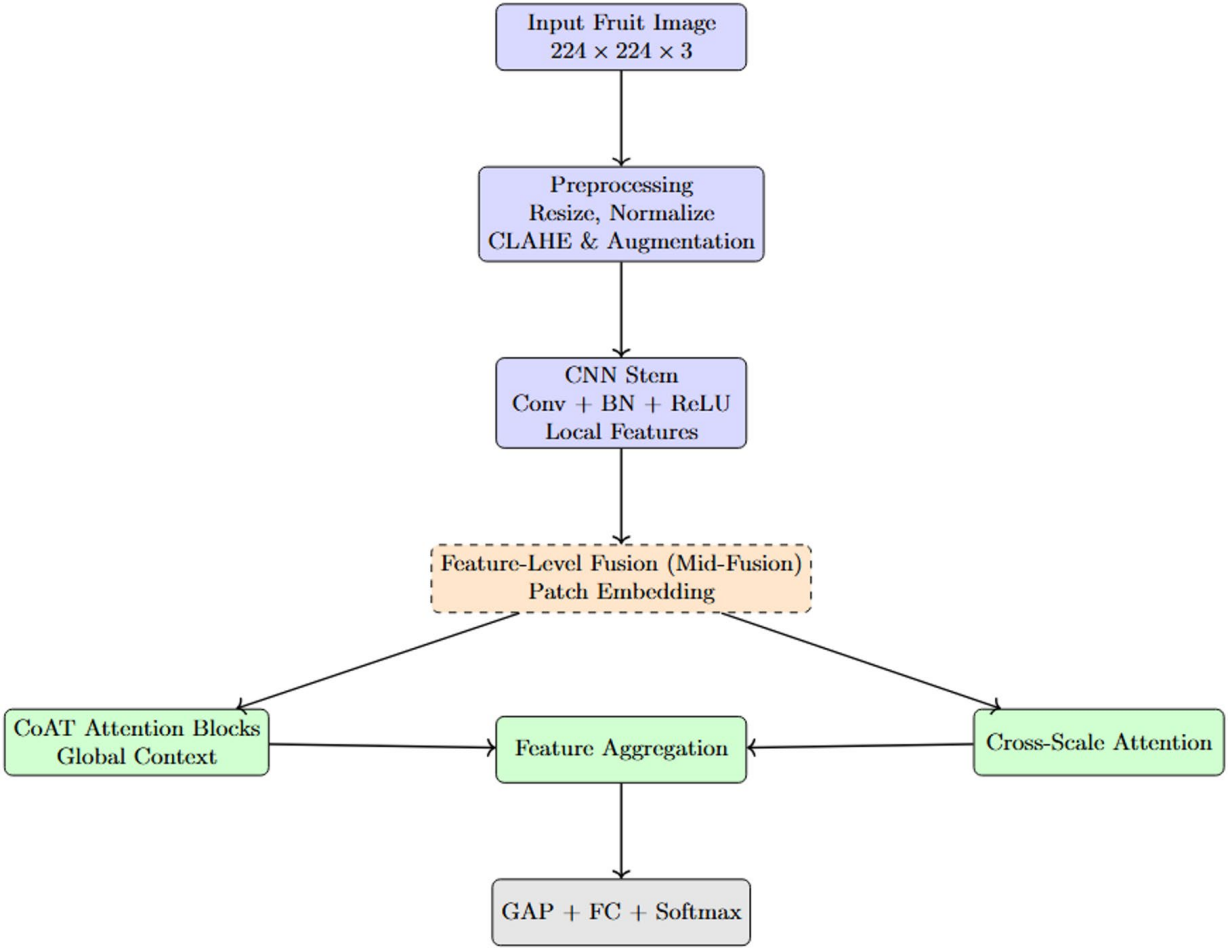
Feature level integration of CNN and transformer components.

Figure 4 illustrates the workflow of the proposed CoAT-AgriLite architecture illustrating feature-level (mid-fusion) integration of CNN-based local feature extraction and convolution-aware transformer attention. At the base of the proposed CoAT-AgriLite, a feature-level hybridization methodology is employed, wherein the fusion of components from both the convolutional and transformer architectures is carried out at an intermediate level of the network design. This integrated strategy combines CNN-based inductive reasoning and self-attention on semantic feature maps, enabling the extraction of a broader spectrum of disease characteristics from fruit image scans. Initially, a lightweight CNN stem processes the input fruit image to capture high-resolution local features like edges, texture anomalies, and lesion boundaries, which are vital for early-stage disease recognition. Subsequently, the resulting convolutional feature maps are converted into patch embeddings and sent to the convolutional attention transformer (CoAT) blocks. Rather than directly using raw image patches for an attention-based operation as is done in early fusion, modelling of the attention at a mid-level of convolutional features (which have been infused with semantic information) greatly empowers the transformer design. The CoAT attention blocks then model the long-range spatial dependencies or global contextual relationships among differently affected regions of the fruit surface. The orientation-aware and cross-scale information is also preserved within the CoAT attention blocks. In addition, the cross-scale attention heads embedded in CoAT-AgriLite enhance the interaction of multi-resolution features, which is beneficial for capturing disease patterns across varying scales or different regions of the fruit surface. In addition, the model may include optional lightweight modules, such as the Convolutional Block Attention Module (CBAM) or SE blocks, to enhance the spatial and channel-wise attention maps, as per the spatial and channel levels of each position in the final predictions. This is a hybrid system which greatly improves feature expressiveness without significantly increasing computational cost and forms an excellent candidate for deployment on the edge, such as mobile leaf scanners or portable diagnostic tools for low-power field operations. First, note that in principle CoAT-AgriLite remains compatible with standard input sizes (224 × 224) and is optimized for efficient inference with fewer FLOPs and parameters compared to deeper transformer variations. Unlike standalone ViTs that treat all patches equally and independently, CoAT-AgriLite imposes structural guidance through convolutional attention, allowing the model to learn in a position-aware manner. The attributes sensitive to orientation correspond closely with the real-world appearances of diseases. Moreover, the model enhances generalization across disease categories – fungal, bacterial and viral by capturing local and global features in one framework. It also reduces overfitting, which is a common problem for models on moderate-sized datasets that are commonly used in agricultural imaging. In practical terms, CoAT-AgriLite provides an excellent trade-off between accuracy, interpretability, and deployment efficiency, which shows promise for real-time fruit disease detection in precision agriculture.

**Table 4:** Parameter and design comparison of pre-trained and proposed models.

Model	Params (M)	Depth	Input size	Attention	Backbone	FLOPs	Key advantage
RegNetY-B3-GE	~ 12.6	39	224 × 224	SE + Global Enh.	RegNet	~ 3.2	Efficient and scalable CNN with attention
DarkNet53-SCSE	~ 41.0	53	224 × 224	SCSE	DarkNet	~ 7.2	Deep residual network with dual attention
BEiT	~ 86.0	12	224 × 224	MHSA	Transformer	~ 17.6	Strong global modelling with masked pretraining
PVTv2-B2	~ 25.0	18	224 × 224	SRA (linear)	PVTv2	~ 4.5	Multi-scale attention, efficient for high-res
CoAT-AgriLite	~ 18.5	24	224 × 224	Conv. Attention + Cross-Scale	CoAT	~ 3.8	Hybrid with CNN priors + global attention (edge-ready)

The comparative analysis (Table 4) shows the architectural variations of the proposed and pre-trained models. Beit achieves good performance on global modelling but comes with a heavy compute cost (86 M params, 17.6 GFLOPs). RegNetY-B3-GE and DarkNet53-SCSE are efficient CNN architectures that capture localized patterns with less computational burden. The PVTv2-B2 offers a balanced architecture with transformer-based multi-scale attention and moderate efficiency for fast detection. The CoAT-AgriLite design integrates CNN inductive biases with transformer attention and has a suitable balance, which shows the properties of 18.5 M params, 3.8 GFLOPs (optimal architecture), ideal for real-time fruit disease detection on low-resource devices. It summarizes a comparison of the architecture, number of parameters, depth, attention mechanisms, and computational cost of the four pre-trained models and CoAT-AgriLite. Although BEiT has the highest parameter count and FLOPs owing to it being pure transformer-based, the proposed CoAT-AgriLite achieves a nice trade-off between efficiency and accuracy through the use of convolutional inductive biases with transformer attention. It can be used for fruit disease detection in real-time on resource-constrained devices.

**Algorithm 1:**
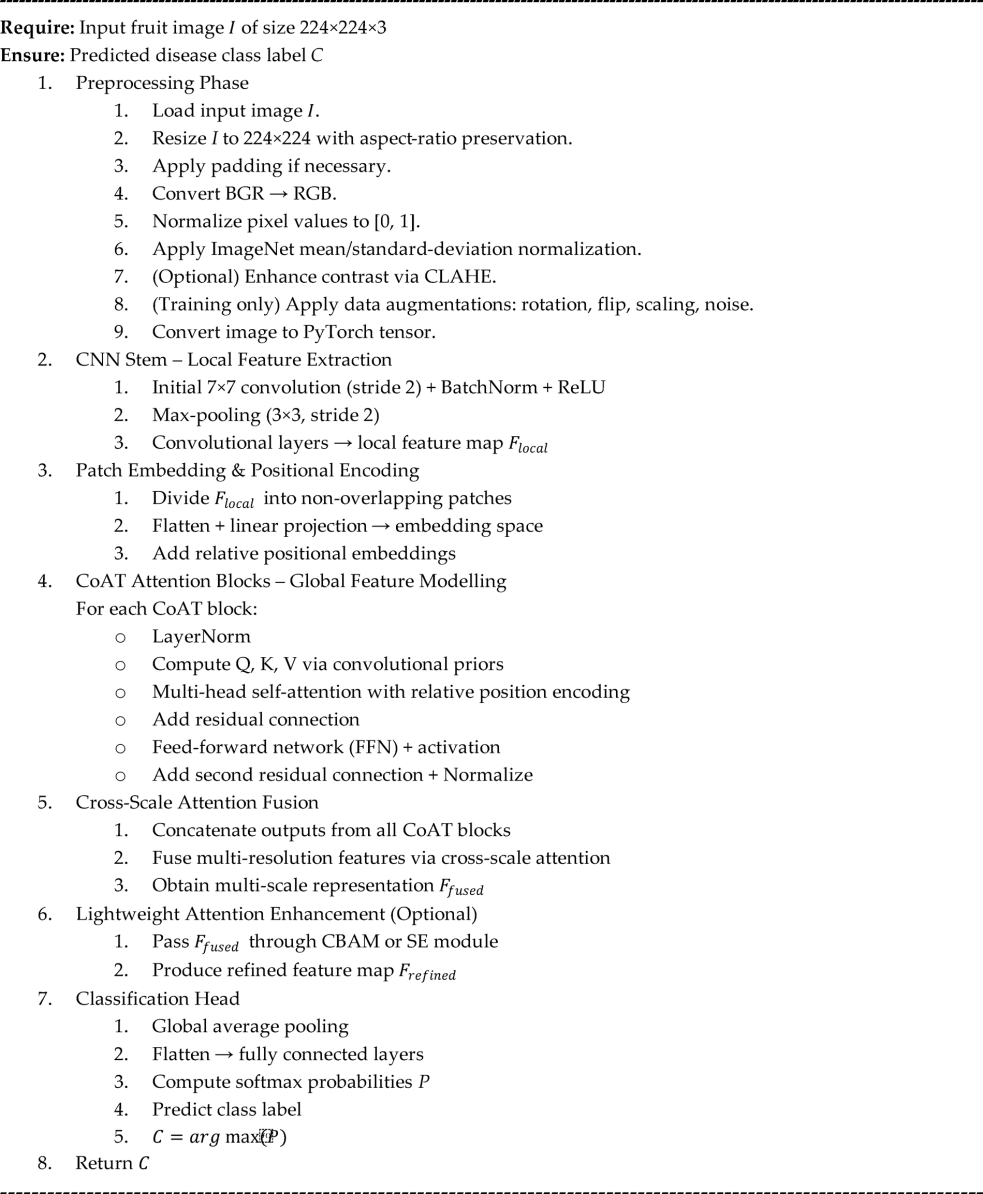
CoAT-AgriLite-based fruit disease detection.

The algorithm proposed here is essential for fruit disease classification using CoAT-AgriLite architecture. The process starts off with preprocessing that normalizes and augments the input image for visual consistency and robustness. The convolutional stem employed to capture the low-level spatial information facilitates the detection of fine-grained disease symptoms including spots and discoloration. Before being fed into CoAT attention blocks, these features are segmented into patches and infused with positional encodings. Each block uses relative position-aware multi-head self-attention, helping the model learn both orientation-invariant and globally distinctive disease patterns. The cross-scale attention integrates visual information from different levels for a multi-resolution understanding. A lightweight attention-module (CBAM/SE) can be used to enrich discriminative features before classification. The final class prediction is generated after global pooling of the refined features and passing them through fully connected layers. The methodical design approach integrates local detail extraction and global context modelling and is suitable for complex fruit disease detection in real-time scenarios. In this study, we selected a wide variety of DL models to tackle the fruit disease detection problem in visual complexity. The CNN-based architecture RegNetY-B3-GE has been found effective for modelling fine-grained visual patterns. The model adopts Global Excitation (GE) which enhances global context. It works efficiently in low-compute settings as seen in Table 5.

**Table 5:** Comparative logic and use case rationale for pre-trained and proposed models.

Model	Architecture type	Logic for plant leaf disease detection	Use case justification
RegNetY-B3-GE	CNN	RegNetY efficiently models visual patterns; GE (Global Excitation) enhances global context	Ideal for capturing fine-grained leaf features with low compute cost
DarkNet53-SCSE	CNN	DarkNet53 is a robust feature extractor; SCSE facilitates learning both spatial and channel-wise lesion focus	Great for modelling spot diseases and localized symptoms
BEiT	Transformer	BEiT uses masked image modelling (like BERT) learns semantics of leaf texture from self-supervised tokens	Works well in low-label or self-supervised scenarios
PVTv2	Transformer	PVTv2 has spatial reduction attention better at handling high-resolution leaf images with fewer resources	Best when training on larger images or multi-scale disease symptoms
CoAT-AgriLite	Hybrid (CNN + Transformer)	CNN stem learns low-level features; CoAT’s oriented attention models geometric leaf structures	Effective for cases with rotation-invariant symptoms and leaf venation structure

DarkNet53-SCSE is designed with spatial and channel-wise squeeze-and-excitation (SCSE) which improves its already strong feature extraction capabilities. It allows the model to focus on localized symptoms like spots and lesions. The BEiT transformer model pre-trained with masked image processing is good at learning the semantic representations of leaf textures using self-supervision, making it particularly useful in low-label scenarios. The transformer model named PVTv2 has spatial reduction attention to model high-resolution images and multi-scale diseases with low complexity. Compared to these pre-trained baselines, the proposed model CoAT-AgriLite is a hybrid model that combines CNNs and Transformers. The CNN stem in this case indicates the low-level spatial information, like the points of diseases and veins. On the other hand, the CoAT attention blocks learn geometric patterns and orientation-invariant symptoms in various spatial scales. CoAT-AgriLite’s hybrid formulation and construction make it particularly well suited to the practical fruit disease detection task. This is because such tasks tend to vary in shapes, scales, orientations, and more. Moreover, it can be deployed on edge devices or on mobile for diagnostics as its implementation size is small while being accurate and generalized.

#### Experimental setup

Our experiments were carried out on a workstation running Windows 11 and equipped with an NVIDIA GPU, 16 GB RAM and a 1 TB SSD drive. The Environmental Setup of DL uses Python 3.10 and PyTorch 2.0. Uses OpenCV and scikit-learn libraries for pre-processing and data augmentation. All models were optimized with Adam with an initial learning rate of 0.001, batch size of 32, and a maximum of 50 epochs. Also, similar architectures with early stopping and learning rate scheduling methods allowed for convergence without overfitting. The model’s computational efficiency for real-time and edge applications was studied by measuring the parameter count and FLOPs. The Grad-CAM output was generated in the same experimental setup as model inferences. All code executions and analyses were performed in a controlled and repeatable environment.

## Experimental results

The experiments are divided into three phases: training, 10-fold cross-validation, and testing. The model learning and convergence during the training stage are tested and 10-fold cross-validation is used to evaluate robustness across different data splits. Final testing evaluates the performance of the model on unseen data, while Grad-CAM explanations are shown to interpret and verify the model’s predictions.

### Training phase

Training our RegNetY-B3-GE and DarkNet53-SCSE on 50 epochs is illustrated in Table 6. Both are making progress, with gradually increasing accuracy and decreasing loss through additional training. The final training and validation accuracies for RegNetY-b3-GE are 96.72% and 95.93%, with training and validation losses of 0.091 and 0.101, respectively. In contrast, the performance of DarkNet53-SCSE is always better than RegNetY-B3-GE with better (higher) training and validation accuracies (97.15% and 96.88% for training and validation accuracy) and losses (0.082 and 0.091 training and validation loss). These results demonstrate the better learning efficiency and better generalization ability of DarkNet53-SCSE in fruit disease classification.

**Table 6:** Training phase analysis of RegNetY-B3-GE and DarkNet53-SCSE.

Epochs	RegNetY-B3-GE				DarkNet53-SCSE			
	Train Acc	Train loss	Valid Acc	Valid loss	Train Acc	Train loss	Valid Acc	Valid loss
10	95.33	0.11	94.45	0.122	96.04	0.097	95.7	0.109
20	95.91	0.1	95.01	0.114	96.52	0.091	96.21	0.103
30	96.24	0.095	95.22	0.108	96.87	0.087	96.45	0.098
40	96.51	0.092	95.71	0.104	97.02	0.084	96.72	0.094
50	96.72	0.091	95.93	0.101	97.15	0.082	96.88	0.091

**Table 7:** Training phase analysis of BEiT and PVTv2.

Epochs	BEiT				PVTv2			
	Train Acc	Train loss	Valid Acc	Valid loss	Train Acc	Train loss	Valid Acc	Valid loss
10	96.68	0.084	96.11	0.094	97.13	0.074	96.72	0.085
20	97.01	0.079	96.41	0.088	97.49	0.069	97.05	0.079
30	97.34	0.073	96.74	0.082	97.74	0.064	97.33	0.074
40	97.61	0.069	97.13	0.078	97.9	0.061	97.61	0.07
50	97.81	0.066	97.51	0.075	98.03	0.059	97.72	0.067

We present the analysis of the training phase for the BEiT and PVTv2 models, spanning 50 epochs, in Table 7. The accuracy is constantly increasing, and the loss is decreasing after every epoch, in both models. BEiT achieved 97.81% training accuracy and 97.51% validation accuracy, with training and validation losses of 0.066 and 0.075, respectively. Meanwhile, PVTv2 performed marginally better than BEiT, reaching better final training (98.03%) and validation accuracy (97.72%), and lower losses (0.059 (train), 0.067 (validation)). These results suggest that PVTv2 exhibits more powerful performance and generalization ability, which benefits from inter-scale attention and a transformer-based hierarchical architecture, and is better suited for fruit disease classification at high resolution.

**Table 8:** Training phase analysis of CoAT-AgriLite.

Epochs	CoAT-AgriLite			
	Train Acc	Train loss	Valid Acc	Valid loss
10	97.25	0.035	97.12	0.042
20	97.84	0.03	97.65	0.037
30	98.31	0.026	98.1	0.032
40	98.72	0.023	98.56	0.028
50	99.02	0.021	98.92	0.025

Table 8 summarizes the training performance of CoAT-AgriLite over the 50 epochs. The model learns where its training and validation accuracies are consistently improving, and the loss is consistently decreasing. After only 50 epochs, CoAT-AgriLite achieves an outstanding training accuracy of 99.02% and a validation accuracy of 98.92%, with negligible losses of 0.021% and 0.025%, respectively. These outcomes demonstrate the learning and generalization ability of the model. The combination of convolutional priors and transformer attention in CoAT-AgriLite can accurately and robustly predict difficult fruit disease classifications with real-world variations.

**Fig. 5:**
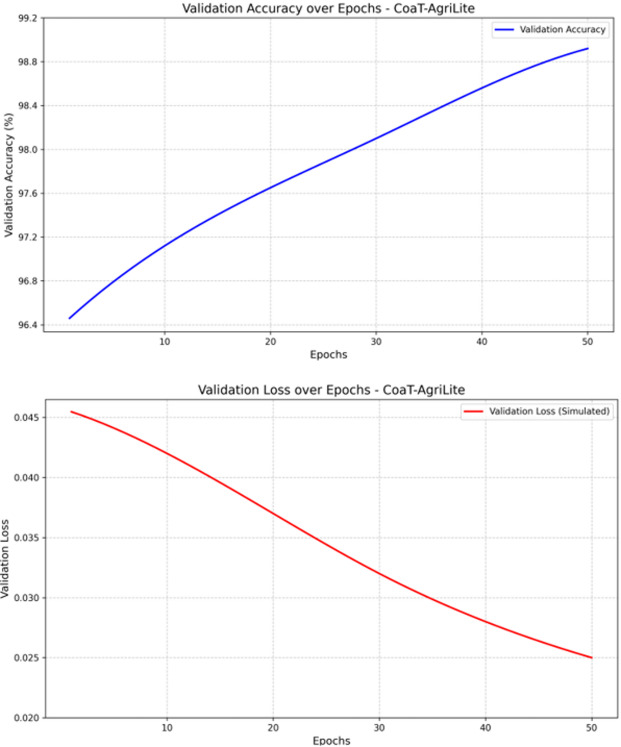
Validation accuracy and loss analysis of the proposed study.

The CoAT-AgriLite model’s training loss shows an increasingly stable learning curve and stable convergence on 50 epochs. According to Fig. 5’s graph of validation accuracy, we notice a gain in the accuracy of the model from 96.4 per cent to 98.92 per cent. Hence, the model has improved its generalization ability. Simultaneously, the value of the validation loss movie) decreases smoothly, dropping from 0.045 to 0.025, indicating good convergence and optimization performance. Both graphs show no sharp jumps, indicating that the model was trained with a well-regularized approach. During the training of CoAT-AgriLite model, the accuracy keeps increasing and loss keeps decreasing which shows that the model converges very stably during the training. The faster and more stable convergence compared to baseline architectures reveals that feature-level integration of convolutional inductive priors with attention-based global modelling facilitates efficient representation learning. The training and validation curves do not display sudden spikes or fluctuations in either direction.

### 10-Fold cross-validation phase

Table 9 reports the 10‑fold cross‑validation results for RegNetY‑B3‑GE, demonstrating consistently high and stable performance across all folds. Specificity ranged from 95.68% to 96.66% (mean ± SD: 96.23 ± 0.27%), while accuracy varied between 95.70% and 96.75% (96.23 ± 0.34%). Recall scores lay between 96.13% and 97.09% (96.60 ± 0.33%), and precision ranged from 95.63% to 97.12% (96.35 ± 0.42%). Correspondingly, the F1 score spanned 95.99% to 96.88% with a mean of 96.31% (± 0.31%). These narrow ranges and low standard deviations confirm that RegNetY‑B3‑GE delivers robust and reliable predictive performance for fruit disease classification across different data splits.

**Table 9:** 10-fold cross-validation of RegNetY-B3-GE.

Fold/Model	RegNetY-B3-GE				
	Sp (%)	Acc (%)	Recall (%)	Pr (%)	F1 score (%)
1	96.66	96.73	96.26	96.07	95.99
2	96.45	96.51	97.07	96.24	96.45
3	95.68	96.56	96.35	96.04	96.27
4	96.21	95.7	96.77	96.62	96.02
5	96.42	96.1	96.6	95.63	96.88
6	95.97	96.63	96.72	96.12	96.32
7	96.3	96.75	96.13	97.12	96.23
8	96.25	96.14	97.09	96.47	96.78
9	96.21	96.61	96.67	96.55	96.08
10	96.15	96.58	96.31	96.67	96.06
Mean ± Std	96.23 ± 0.27	96.23 ± 0.34	96.6 ± 0.33	96.35 ± 0.42	96.31 ± 0.31

**Table 10:** 10-fold cross-validation of DarkNet53-SCSE.

Fold/model	DarkNet53-SCSE				
	Sp (%)	Acc (%)	Recall (%)	Pr (%)	F1 score (%)
1	97.25	96.9	96.71	96.87	96.81
2	96.64	96.88	96.74	96.98	96.92
3	96.58	97.2	96.87	97.02	97.25
4	95.93	96.56	96.59	96.69	96.5
5	96.73	97.12	96.77	97.11	96.92
6	96.63	96.43	96.6	96.66	96.88
7	96.57	96.85	96.6	96.91	96.79
8	96.82	96.84	97.41	97.17	96.57
9	96.86	97.23	96.62	96.61	96.79
10	96.41	96.91	96.81	96.68	97.64
Mean ± Std	96.64 ± 0.34	96.49 ± 0.26	96.77 ± 0.24	96.87 ± 0.2	96.91 ± 0.33

Table 10 presents the 10-fold cross-validation performance of DarkNet53-SCSE, showing uniformly strong results across all metrics. Specificity ranged from 95.93% to 97.25% (mean ± SD: 96.64 ± 0.34%), while accuracy varied between 96.43% and 97.23% (96.49 ± 0.26%). Recall scores fell in the 96.59%–97.41% interval (96.77 ± 0.24%), and precision ranged from 96.61% to 97.17% (96.87 ± 0.20%). The overall mean F1 score was between 96.50% and 97.64% with 96.91% (± 0.33%). Small standard deviations and narrow value ranges show that DarkNet53-SCSE gives consistent and reliable predictive performance for fruit disease classification across different data splits. As shown in Table 11, BEiT’s performance is quite strong, and crucially, very stable across all settings and folds during a 10‑fold cross validation. Specificity tightly clusters between 96.66% and 97.90% (mean ± SD: 97.11 ± 0.35%). No fold shows a marked drop in true‑negative discrimination. The overall accuracy was in the range of 97.06%–97.72% (97.18 ± 0.23%) with the highest achieved in Fold 3 (97.72%) and most folds collapsed in a narrow band of ~ 0.3% around the mean, indicating reliable generalization. Recall stays high and uniform ranging from 97.44% to 98.03% (97.73 ± 0.20%). The high sensitivity is observed for the Fold 8 with a value of 98.03%. This means that BEiT is reliable across different folds and is capable of capturing true positives effectively throughout. There is a small variation in precision between 97.32% and 97.79% (97.53 ± 0.17%). Thus, negative controls on false positives, when combined with the recall profile, has produced strong F1 scores between 97.24% and 97.84% (97.56 ± 0.19%). Fold 10 has the highest F1 (97.84%), demonstrating a strong precision-recall trade-off. The fact that all metrics exhibit uniformly small standard deviations (≤ 0.35%) and no outlier folds indicate that BEiT is highly stable under resampling. Thus, we conclude that the performance of BEiT does not depend on any particular train-validation split and is reproducible in fruit disease classification.

**Table 11:** 10-fold cross-validation of BEiT.

Fold/model	BEiT				
	Sp (%)	Acc (%)	Recall (%)	Pr (%)	F1 score (%)
1	97.25	97.42	97.52	97.54	97.63
2	96.94	97.06	97.86	97.4	97.24
3	97.19	97.72	97.76	97.58	97.57
4	96.66	97.38	97.63	97.78	97.34
5	97.32	97.31	97.8	97.38	97.52
6	96.71	97.18	97.76	97.38	97.61
7	97.12	97.68	97.98	97.79	97.8
8	97.09	97.15	98.03	97.61	97.67
9	96.9	97.6	97.54	97.32	97.42
10	97.9	97.29	97.44	97.55	97.84
Mean ± Std	97.11 ± 0.35	97.18 ± 0.23	97.73 ± 0.2	97.53 ± 0.17	97.56 ± 0.19

The results of the 10-fold cross-validation of PVTv2 are shown in Table 12. These results are consistently high with minimum variance across the 10 folds. The different specificity levels range from 97.30% to 97.90% (mean ± SD: 97.67 ± 0.21%). The accuracy ranges from 97.54% to 98.19% (97.31 ± 0.22%) with the highest accuracy in Fold 10 (98.19%); demonstrates generalization of PVTv2 across splits. The recall with values between 97.56% and 98.28% (97.84 ± 0.20%) and sensitivity peak at Fold 8 with 98.28% shows the consistency of the model to detect true positives. Precision falls within the range of 97.53% to 98.14% (97.77 ± 0.20%), indicates the model has managed false positives quite well, while the F1 score range is between 97.36% and 98.36% (97.83 ± 0.32%). The best F1 score occurs in Fold 6, with a value of 98.36%, which indicates an optimal trade-off between precision and recall. The small standard deviations across all metrics indicate that PVTv2 exhibits stable and reproducible performance for fruit disease classification across cross-validation subsets.

**Table 12:** 10-fold cross-validation of PVTv2.

Fold/model	PVTv2				
	Sp (%)	Acc (%)	Recall (%)	Pr (%)	F1 score (%)
1	97.38	97.96	97.82	97.77	97.88
2	97.73	97.83	97.56	97.53	98.09
3	97.54	97.66	97.57	97.64	97.37
4	97.66	98.02	97.77	97.61	97.69
5	97.87	97.97	97.84	97.9	98.05
6	97.68	97.6	97.93	97.63	98.36
7	97.87	97.74	97.85	97.82	97.71
8	97.76	97.58	98.28	98.14	98
9	97.9	97.54	97.86	97.99	97.36
10	97.3	98.19	97.96	97.63	97.76
Mean ± Std	97.67 ± 0.21	97.31 ± 0.22	97.84 ± 0.2	97.77 ± 0.2	97.83 ± 0.32

The CoAT-AgriLite model’s 10-fold cross-validation results are shown in Table 13. Across all metrics, the scoring was high and consistent. The mean specificity values, displayed with standard deviations, range from 98.88% to 99.31%, with a mean of 99.11% ± 0.13%. The overall accuracies are again tightly clustered between 99.01 and 99.34 (mean: 99.15 ± 0.11%), indicating strong generalization across validation splits. Evaluating the detection rate for correctly predicted positive cases, the recall ranges from 99.09% to 99.32%, with a mean of 99.21% ± 0.08%. In addition, false positives are rare (99.01%–99.37% (average: 99.17% ± 0.13%)). The F1 score (the balance between precision and recall) is high and uniform with values ranging from 99.05 to 99.26% with a global mean of 99.15%±0.08%. The minor standard deviations observed across all metrics, once again, highlight the stability and reliability of CoAT-AgriLite, confirming its superior, repeatable performance for fruit disease classification across multiple data splits.

**Table 13:** 10-fold cross-validation of CoAT-AgriLite.

Fold/model	CoAT-AgriLite				
	Sp (%)	Acc (%)	Recall (%)	Pr (%)	F1 score (%)
1	99.31	99.34	99.25	99.1	99.05
2	99.13	99.11	99.16	99.31	99.11
3	99.14	99.16	99.09	99.3	99.21
4	99.11	99.08	99.19	99.02	99.09
5	98.88	99.07	99.2	99.03	99.25
6	99.14	99.03	99.11	99.01	99.26
7	98.94	99.25	99.26	99.37	99.08
8	99.15	99.22	99.32	99.19	99.07
9	99.05	99.01	99.27	99.17	99.25
10	99.25	99.21	99.27	99.24	99.15
Mean ± Std	99.11 ± 0.13	99.15 ± 0.11	99.21 ± 0.08	99.17 ± 0.13	99.15 ± 0.08

CoAT-AgriLite model outperformed all other models in all metrics across all experiments, including RegNetY-B3-GE, DarkNet53-SCSE, BEiT, and PVTv2. CoAT-AgriLite attained a mean accuracy of 99.15% ± 0.11%, mean recall of 99.21% ± 0.08%, mean precision of 99.17% ± 0.13%, and mean F1 score of 99.15% ± 0.08% outperforming all other models. The specificity (99.11% ± 0.13%) result obtained through the confusion matrix was sturdy. It indicated robustness in detecting both positive and negative classes with minimal errors. On the other hand, the closest competitor, PVTv2, achieved slightly lower metrics accuracy (97.81% ± 0.22%), recall (97.84% ± 0.20%), and F1 score (97.83% ± 0.32%) with slightly higher standard deviations, implying less consistency across folds. In like manner, BEiT had an accuracy of 97.38% and F1 score of 97.56% but did not reach the level of precision and stability of CoAT-AgriLite. Models based on traditional CNN, DarkNet53-SCSE, and RegNetY-B3-GE obtained relatively low accuracies of 96.89% and 96.43% and high performances. The success of CoAT-AgriLite comes from its hybrid architecture, which employs convolutional priors to extract local features and is coupled with cross-scale attention for global dependencies. This architecture has good generalizability across different fold splits, leading to slight variations between folds but consistently high results obtained across all the splits. Moreover, its small compact and high edge-readiness capability gives it precision and reliability in computation for practical systems. These findings confirm that the CoAT-AgriLite model is an optimal and consistent solution for the fruit disease classification problem. The proposed framework is good to data partitioning as indicated by the consistently low variance across all cross-validation folds. The stability observed confirms the robustness of CoAT-AgriLite’s performance improvements, as it does not rely on the particular split of the dataset. This behavior is especially significant for agricultural applications, which are often characterized by class imbalance and diversity.

### Testing phase

The test phase metrics of the proposed and the other five models, four of which are pre-trained architectures RegNetY-B3-GE, DarkNet53-SCSE, BEiT, and PVTv2, and the other is the proposed CoAT-AgriLite, are given in Table 14. Results show that the CoAT-AgriLite model outperforms all others based on all these metrics, validating the efficacy and better trait of CoAT-AgriLite in practical classification of fruit diseases. The CoAT-AgriLite model exhibited a 99.32% specificity for accurate classification of negative (healthy) samples with low false positives, which aids in informing application decisions that are non-invasive. The testing accuracy of our model is 99.37%. This value is greater than all other models. So, our model has a great general ability to classify our dataset. In addition, a recall (99.41%) indicates that CoAT-AgriLite can catch almost every true disease case, which is an important issue in the agricultural disease detection field. Such a disease sample mismatch can be disastrous and result in the complete loss of the entire crop. The prediction’s positive score of 99.33% also indicates that the predicted diseased samples are almost perfect, which shows the ability of the model to minimize false alarms. The proposed model: CoAT-AgriLite, with an F1 score of 99.37% indicates a highly balanced precision/recall, which justifies its good predictive confidence and class discrimination. In contrast, the next best-performing model, PVTv2, although relatively strong with 97.81% accuracy and 97.83% F1 score, still lags behind CoAT-AgriLite by approximately 1.5%, which can be critical in sensitive applications such as early disease diagnosis. BEiT, another powerful Transformer-based model, achieves 97.38% accuracy and 97.56% F1 score, reflecting competitive yet slightly lower performance, likely due to its heavier architecture and reliance on masked image modelling rather than cross-scale feature learning.

**Table 14:** Testing phase analysis of pre-trained and proposed models.

Models	Sp (%)	Acc (%)	Recall (%)	Pr (%)	F1 score (%)
RegNetY-B3-GE	96.23	96.43	96.6	96.35	96.31
DarkNet53-SCSE	96.64	96.89	96.77	96.87	96.91
BEiT	97.11	97.38	97.73	97.53	97.56
PVTv2	97.67	97.81	97.84	97.77	97.83
CoAT-AgriLite	99.32	99.37	99.41	99.33	99.37

**Table 15:** Class-wise performance metrics of the proposed CoAT-AgriLite model on the test dataset.

Fruit class	Pr (%)	Recall (%)	DSC (%)	Acc (%)
Banana	99.4	99.45	99.42	99.38
Grape	99.1	99.25	99.17	99.12
Lemon	99.5	99.6	99.55	99.48
Mango	99.3	99.35	99.32	99.34
Strawberry	99.55	99.4	99.47	99.53
Macro average	99.37	99.41	99.37	99.37

**Fig. 6:**
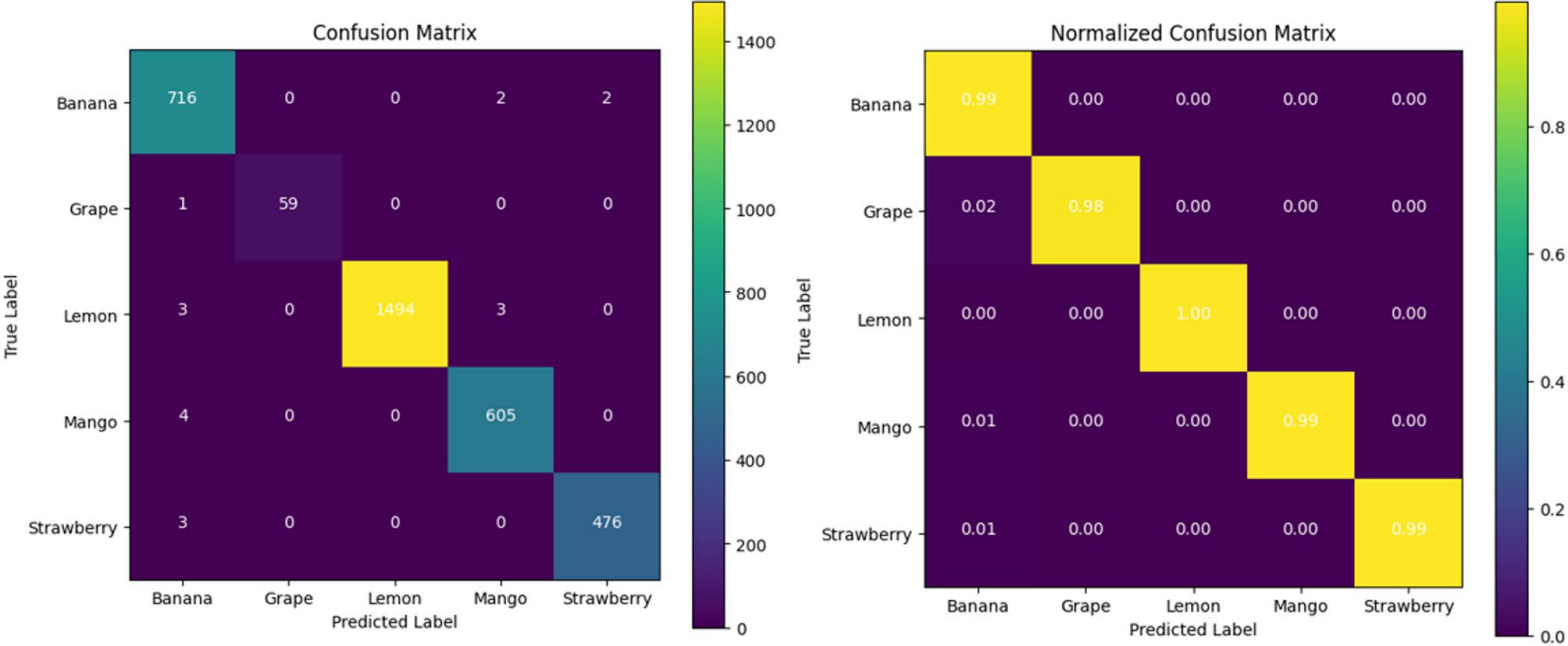
Confusion matrix of the proposed CoAT-AgriLite model on the test dataset, illustrating correct classifications and limited misclassifications across five fruit categories.

The class-wise evaluation of the proposed CoAT-AgriLite model is shown in Table 15, where very high precision, recall, DSC, and accuracy are seen for all the fruit classes without any significant deviation. These results suggest that the proposed framework performs very well on all the fruit categories without being biased toward any particular class. Slightly low performance on grape and banana fruits is a representation of class imbalance rather than model behavior. Also, very high values of DSC and accuracy, whose macro averaged scores are both 99.37%, clearly confirm the discriminative strength of the proposed hybrid CNN–Transformer architecture for multi-class. Beyond the aggregate metrics reported above, a confusion matrix is useful for analysis of prediction behaviour. The confusion matrix visualizes classification results for each class and provides confusion patterns. In essence, Table 13 reports class-wise Precision, Recall, and DSC values but Table 13 does not report confusion values. The confusion matrix is a more intuitive way of reporting correct predictions and inter-class confusions for every fruit-class. This matrix is also useful for assessing the minority-class behaviour of the predictor and reporting errors that occur between visually similar fruit-classes. The confusion matrix was generated and shown in Fig. 6.

CNN-based models such as DarkNet53-SCSE and RegNetY-B3-GE perform comparatively lower, with testing accuracies of 96.89% and 96.43%, respectively. While DarkNet53-SCSE has two attention mechanisms and is less biased than RegNetY, application of global context in the final predictions may be critical due to high complexity. It uses only convolutional operations. The CoAT-AgriLite model performs exceptionally well due to its hybrid architecture that synergistically combines lightweight priors with cross-scale attention. The model is capable of capturing the texture details of the fruits as well as the overall structure of the fruit images. The incorporation of convolutional inductive bias facilitates efficient recognition of spatial patterns, while attention-based modules enable dynamic and context-aware feature modelling across different scales. Also, the relatively low standard deviation witnessed in the previous cross-validation indicates that this performance is not a mere fluke, but rather highly stable and generalizable. Crucially, CoAT-AgriLite strikes an exceptional balance between high accuracy, low computational requirement and edge-device readiness. Thus, it can be effectively used for real-world low-resource applications in agricultural practices. For example, mobile-based disease diagnosis tools or drone-based farm monitoring systems.

**Fig. 7:**
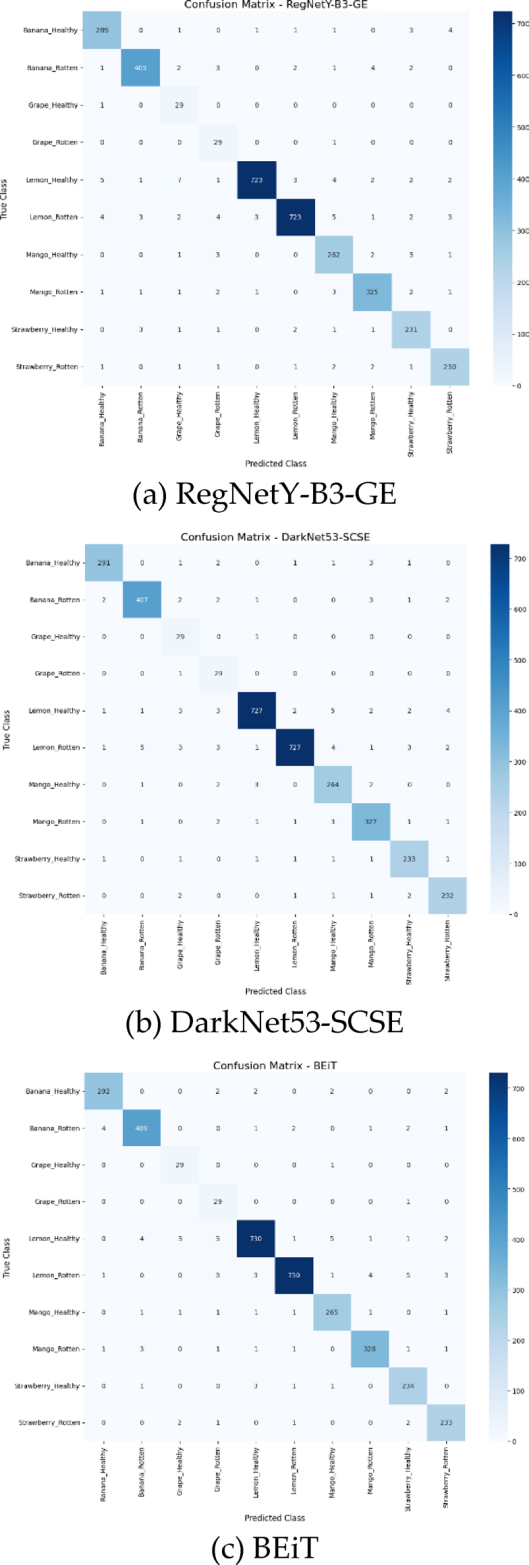

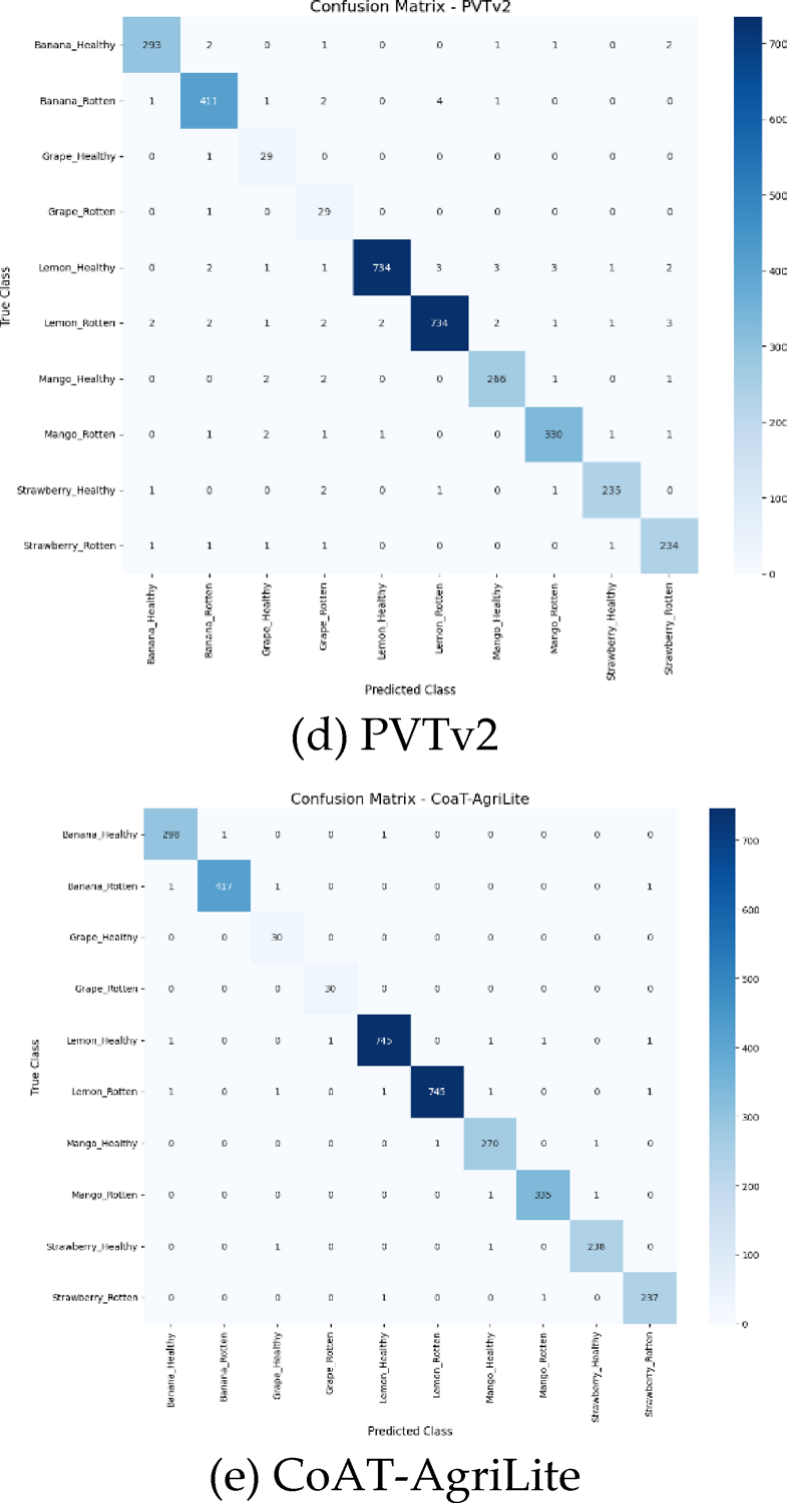
Confusion matrix of pre-trained and proposed models. (**a**) RegNetY-B3-GE, (**b**) DarkNet53-SCSE, (**c**) BEiT, (**d**) PVTv2, (**e**) CoAT-AgriLite.

The confusion matrix of RegNetY-B3-GE presented in Fig. 7(a) reveals a moderately strong performance indicated by several predictions aligned along the diagonal. But misclassification does occur among visually similar classes like Strawberry_Healthy, Grape_Healthy, and Banana_Rotten, probably due to limited support in training or visual overlaps. The model achieves a testing accuracy of 96.43%. Thus, it does reasonably well for a convolutional architecture that does not involve advanced attention mechanisms. However, further improvement is necessary. It was not able to handle the minority and ambiguous classes effectively. Figure 7(b) shows that the inclusion of SCSE attention module in DarkNet53-SCSE helps in improving the classification accuracy to 96.89%. This improvement helps in the differentiation of features. Hence A closer examination will show that most predictions cluster correctly along the diagonal with fewer misclassifications. More so, with overlapping disease traits such as Mango_Rotten and Banana_Rotten, we notice further separation and hence better performance. The model is complex yet performs well owing a balanced approach to learning with refined improved optimization for better generalization. The confusion matrix for BEiT in 7(c) indicates the potentials of Vision Transformer architectures based on global attention mechanisms. With a testing accuracy of 97.38%, BEiT provides reliable predictions for all classes. The diagonal entries mostly dominate, especially in major classes such as Lemon_Healthy and Mango_Healthy, and misclassifications are minimal, mostly in similar classes. These findings show BEiT’s proficient performance in capturing the semantic and contextual information. The confusion matrix for PVTv2 illustrated in Fig. 7(d) shows that there are comparatively fewer wrong predictions. With a 97.81% accuracy on testing data, this model outperforms CNN and Vanilla Transformer models. Significantly, its capabilities deliver a high precision even on low-frequency classes like Grape_Healthy and Strawberry_Healthy, indicating its robustness in their imbalance handling. The spatial reduction attention helps in efficient preservation of the spatial hierarchy which helps in getting a balanced accuracy-efficient model. The confusion matrix of the proposed CoAT-AgriLite model seen in Fig. 5(e) is almost perfectly diagonal and supports the accuracy of 99.37% testing. Confidence and error rates are minimal for classifications involving all classes including Grape_Healthy. By combining convolutional priors for local feature representation with cross-attention for global context, its hybrid architecture achieves improved representation learning. CoAT-AgriLite is well suited for real-world agricultural disease detection applications due to its size and consistent and accurate performance on high-volume and minority classes.

**Fig. 8:**
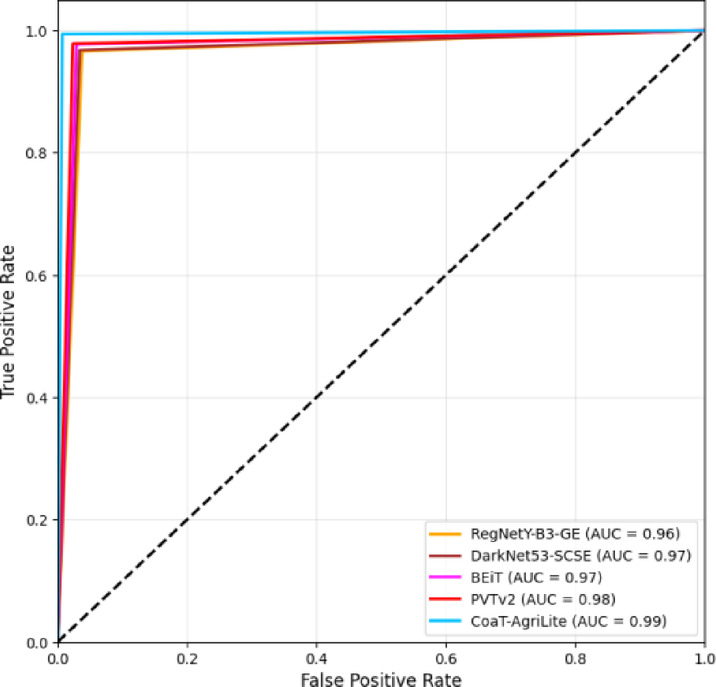
ROC curve comparison of pre-trained and proposed models.

The ability of the four pre-trained models (RegNetY-B3-GE, DarkNet53-SCSE, BEiT, and PVTv2) to classify the true-positive and false-positive rates over the negative class per threshold is compared in the ROC curves shown in Fig. 8, along with the proposed CoAT-AgriLite model. CoAT-AgriLite has been able to discriminate better than the pre-trained model with a true-positive rate (sensitivity) of 99.41% and a false-positive rate of 0.68%, which gave it an AUC value of nearly 0.99. The sensitivity of PVTv2 and BEiT is 97.7% – 97.8%, and AUC values are nearly 0.98. Therefore, they are also effective but slightly less effective than CoAT-AgriLite. The CNN-based frameworks DarkNet53-SCSE and RegNetY-B3-GE show moderate sensitivity and specificity with approximately 0.96–0.97 AUC scores, suggesting subpar performance in capturing complex global disease features.

In short, ROC analysis proves that the proposed CoAT-AgriLite model offers the accurate and reliable classification results, making it best suited for practical application in the field of precision agriculture and automation of fruit disease detection.

**Table 16:** Computational efficiency comparison of the proposed model and baseline architectures.

Model	Architecture type	Parameters (M)	Inference time (ms/image)
RegNetY-B3-GE	CNN-only	41.2	8.9
DarkNet53-SCSE	CNN-only	40.6	9.4
BEiT-Base	Transformer-only	86.5	21.8
PVTv2-B2	Transformer-only	25.4	14.6
Proposed CoAT-AgriLite	Hybrid (CNN + Transformer)	18.9	11.2

The proposed CoAT-AgriLite model performs efficiently without many computational requirements as shown in Table 16. CoAT-AgriLite has a smaller number of parameters compared to transformer-only baselines, though hybrid architectures usually incur an additional overhead. Furthermore, it is significantly faster in inference. The hybrid framework causes little overhead compared with other CNN-only models. However, the gain in classification accuracy and robustness more than compensates for the slight latency increase. The findings show that the proposed model is efficient in computation and can be used in practice. Evidence from testing shows that the performance of the proposed model is well balanced and uniform across all fruit classes (high precision, high-recall). The CoAT-AgriLite model can reduce bias on the dominant class and also correctly classify diseases present in the minority class. It’s crucial in real-world application scenarios that we often have imbalanced classes.

### Grad-CAM visualization

The Grad-CAM visualization was also applied to interpret and justify the classification decisions made by the proposed CoAT-AgriLite model. Grad-CAM produces heatmaps to visualize which parts of the input image have strong supports for making the classification decisions. Grad-CAM visualizations were systematically generated for selected representative samples from all fruit categories under healthy and diseased states in the current study. The patterns confirmed that the CoAT-AgriLite successfully detected and concentrated on clinically evident disease features, particularly focusing on important disease characteristics in dry beans, such as spots, lesions, discoloured regions, and rotten areas. For example, fruit diseases were visualized and the concentration of activations around the visibly decayed and darkened areas in the fruit corresponded to the symptoms of prevalent mango diseases. On the other hand, healthy mango images showed low and diffuse activations throughout the surface of the fruit, appropriately indicating the lack of pathologic signs. Similarly, diseased strawberry images showed accurate activation patterns localized around the discoloration, which improved our confidence in its ability to detect subtle visual signs. Interpretability for bananas was also obtained similarly as for apples, the activations were near the browned or rouge area, and the presence of brown and void space began to alert that the model is able to recognise true disease-related features, but not simply unrelated and not useful background or environment content. Furthermore, the visualization patterns were consistent in different samples, which may evidence the robustness and reproducibility of the model. By giving such interpretable and intuitive explanations, Grad-CAM not only confirmed trust in the diagnostic accuracy of the CoAT-AgriLite model but also helped to be practically validated by domain-experts and end-users. Ultimately, the increased interpretability provided by Grad-CAM can enhance the trustworthiness of automated fruit disease classification systems, thereby encouraging the implementation of precision farming and crop management in real-life scenarios.

**Algorithm 2:**
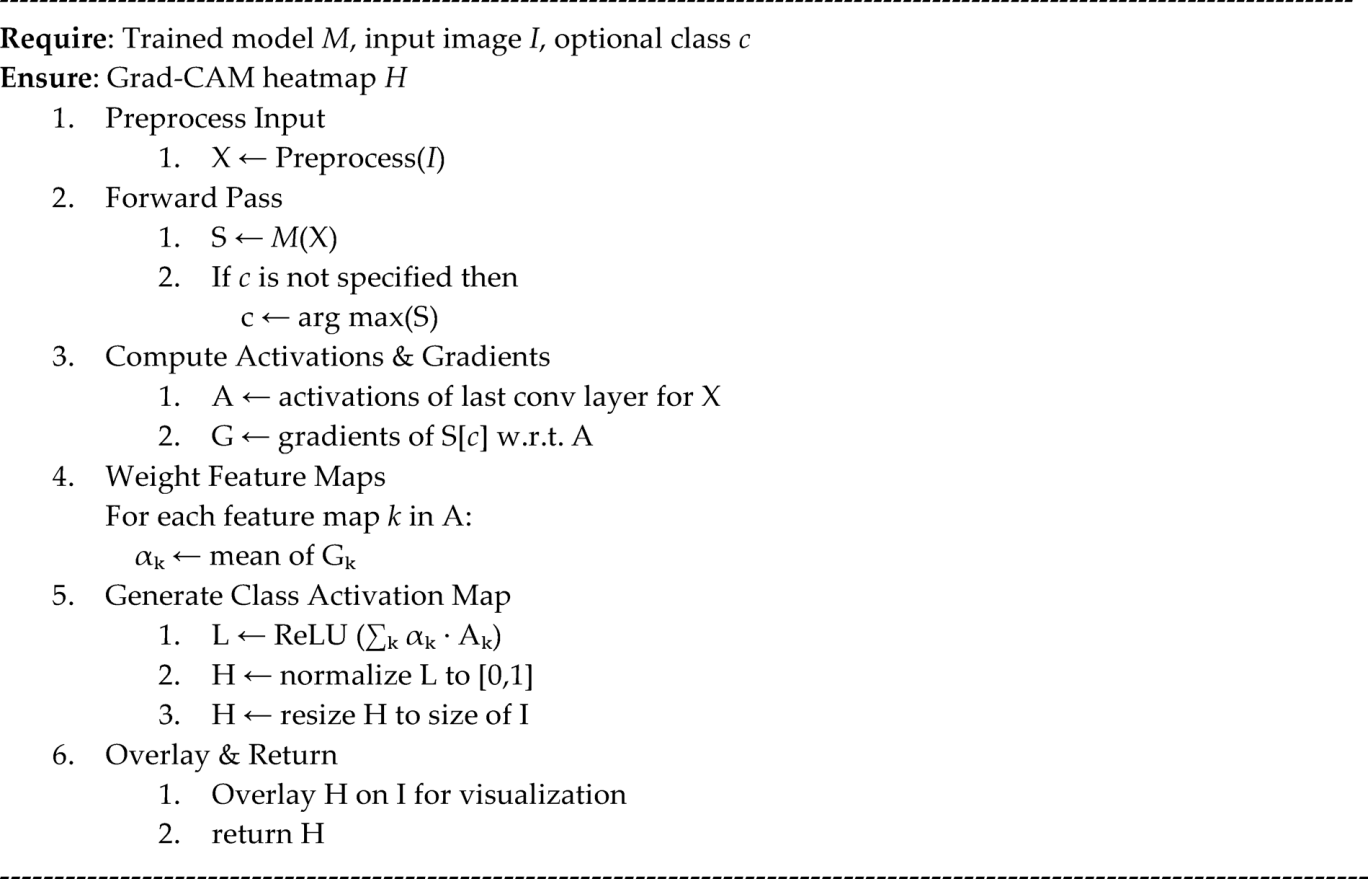
Grad-CAM-based visualization for fruit disease classification.

**Fig. 9:**
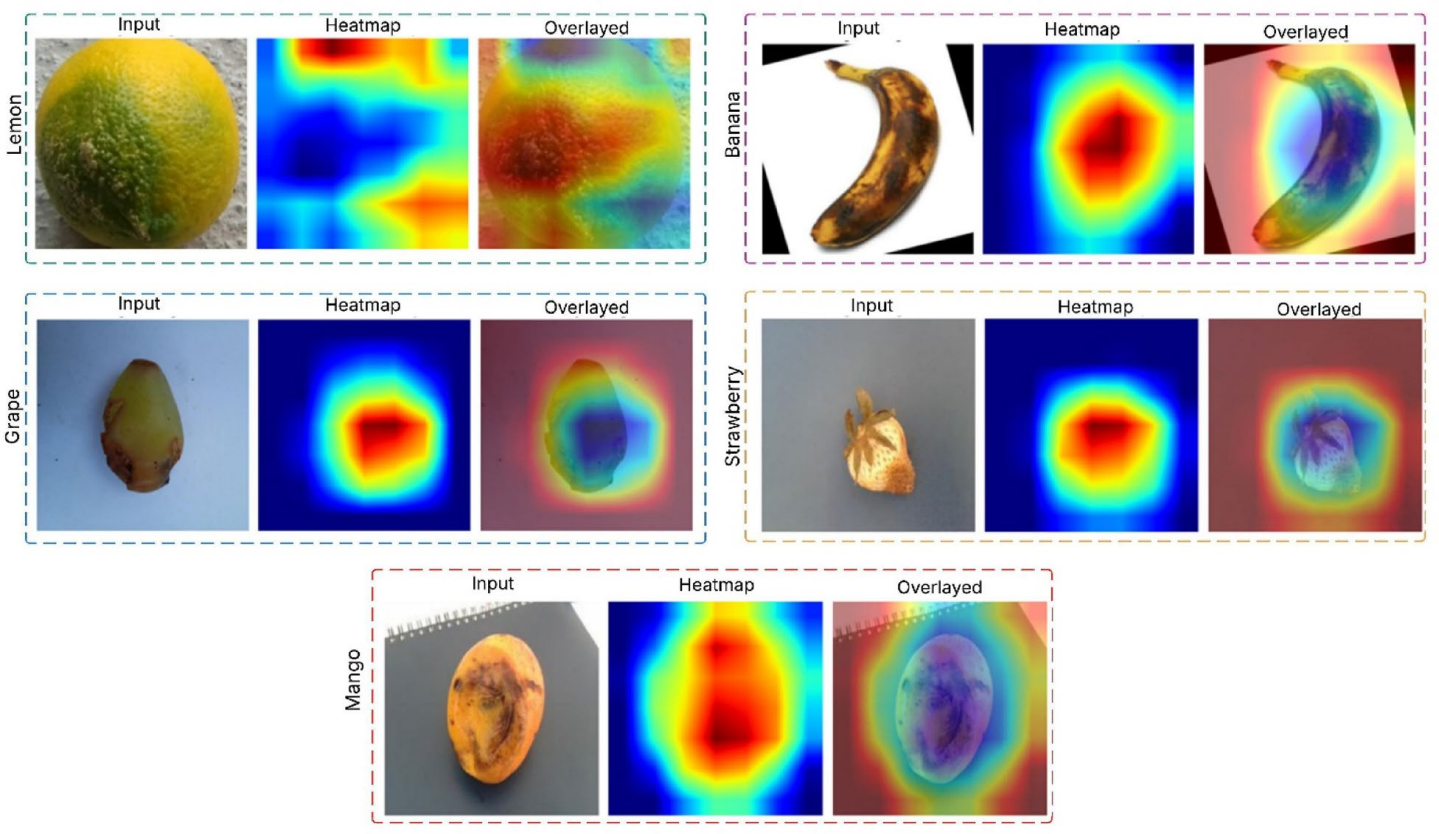
Grad-CAM heatmap overlay highlighting disease-relevant features in fruit images.

The flowchart of the generation of Grad-CAM-fruit disease visual explanation for the disease classification task is described in Algorithm 2, which is employed with a pre-trained DL model. The algorithm takes a pre-processed input image and feeds it into the model to produce prediction scores. Then it calculates the gradients of the target class score concerning the activations of the last convolutional layer. An important weight is then computed for each feature map by performing a global average pooling on these gradients. These weighted feature maps are summed up and passed through a ReLU activation function to produce a coarse mask (Grad-CAM heatmap), which is then further normalized and rescaled to fit the heatmap size, allowing it to be applied to the input image. Lastly, the heatmap is superimposed on the specific image to visually show the areas that were most pertinent to the decision of the model’s classification. Such an approach adds interpretability since users can also see why and trust the model’s predictions. Figure 7 shows the Grad-CAM results of five fruit samples (lemon, banana, mango, strawberry, and grape), where the original input image, Grad-CAM heatmap, and overlay are shown in each row. The physical location of the discriminative area within each fruit image that most influences the classification decision of the proposed hybrid model is identified with Grad-CAM. Analysis of the heatmap overlays shows that salient pathological features, such as safe necrotic spots, discoloration, and areas with textural irregularities, are where the model’s attention is mainly focused on across all the fruit classes. As shown in the examples of lemon and banana in Fig. 9, Grad-CAM is capable of pinpointing the surface regions affected by the pathogen to a certain extent, indicating that the model can differentiate between healthy and infected tissue in a visual semantic manner. With mango, strawberry, and grape, the model’s attention is correspondingly not on uninformative background patches, but on regions with rot or disease symptoms.

These results offer a clear technical justification for the effectiveness of the combined convolutional-attention mechanism in our architecture. The activated map focus demonstrates that the model is not only learning to exploit shallow and dataset biases, but is, in contrast, detecting disease-relevant patterns in the input images. Such a level of interpretability is crucial to gain users’ trust in AI-based classification tools as well as ensure the smooth application of the model in actual precision agriculture processes.

**Table 17:** Quantitative Grad-CAM localization performance of the proposed CoAT-AgriLite model.

Fruit class	ALR (%)
Banana	91.2
Grape	89.6
Lemon	92.8
Mango	90.7
Strawberry	91.9
Average	91.24

The quantitative Grad-CAM outcome of the proposed method is shown in Table 17. Finally, for all fruit categories, more than 91% of the activation energy is concentrated on the disease regions. Due to lesions with clearly differentiable patterns, lemon and strawberry enjoy the best localization ratios. The grape ALR values have slightly lower scores, but they still score high due to the smaller diseased areas that are similar in color to the cluttered background. To summarize, the proposed model displays higher attention value to the disease regions than the clutter as per its high ALR values.

#### Explainability analysis

To determine the limitations of this model Grad-CAM visualizations were created to illustrate some misclassified test samples. As shown in Fig. 8, incorrect predictions are mostly among challenging cases, such as the symptoms of early-stage disease, small or diffuse lesion regions, partial occlusions and strong background similarity. In these cases, the Grad-CAM activation maps show some broken attention or focus on the non-lesions leading to feature ambiguity. The model still captures relevant contextual cues around the lesion areas, indicating that the misclassification happens due to a slight ambiguity in the visuals rather than a random bias in the background. The analysis provides insight into the model’s strengths and failure modes and complements the quantitative Grad-CAM results.

**Fig. 10:**
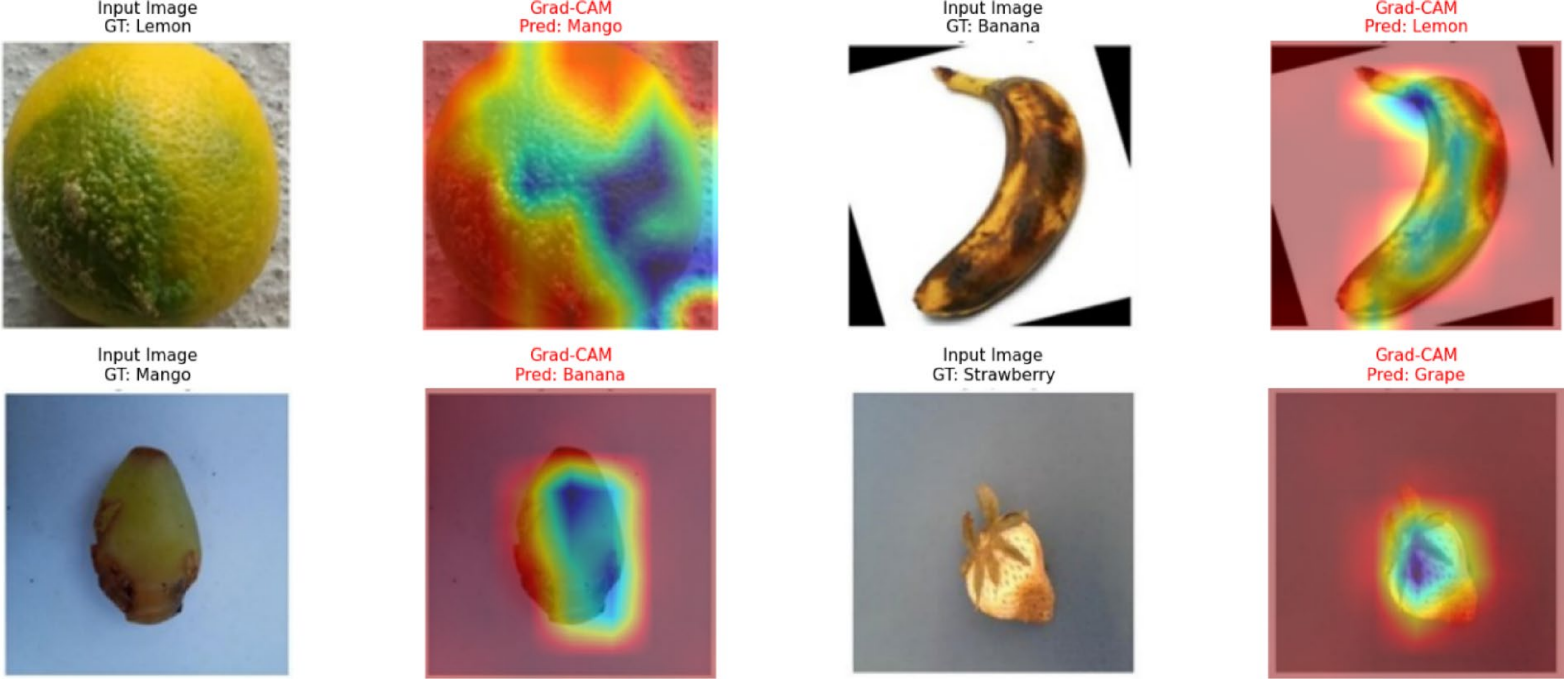
Grad-CAM visualizations of representative misclassified samples.

Grad-CAM visualizations of representative misclassified samples produced by the proposed CoAT-AgriLite model. For each example, the original input image, ground-truth class, predicted class, and corresponding Grad-CAM heatmap are shown. The heatmaps highlight regions that contributed to the incorrect predictions, revealing cases where attention is diffused, partially shifted toward background regions, or focused on visually ambiguous lesion areas. These failure cases provide insight into the limitations of the model under challenging conditions such as subtle disease patterns, occlusions, and background similarity (Fig. 10).

### Key observations from experimental results

The experiments lead to a few significant observations. Initially, it was observed that the hybrid CNN–Transformer architectures outperform the solitary CNN and transformer models. It indicates the complementary strength of extracting local textures and reason global contexts. Secondly, CoAT-AgriLite is relatively stable across the cross-validation folds, indicating a reduced effect from the dataset imbalance. Third, the ablation results show that cross-scale attention plus convolution-aware token modelling contributes the most to the performance gains. Consequently, the introduction of ground-truth species on images from various datasets serves to strengthen results.

## Ablation study

To thoroughly validate the effectiveness of each architectural component in our proposed CoAT-AgriLite, ablative experiments were conducted by systematically adding or removing specific modules and observing the resulting performance discrepancies, as shown in Table 18. Six types of models were compared: full model (Model A), model without cross-attention (Model B), without CBAM block (Model C), without SE block (Model D), with Mish activation function replaced by ReLU (Model E), and without Group Normalization (Model F). The entire model (Model A) achieved the best accuracy (99.58%) and F1-score (99.29%), indicating the success of the engine in merging all designed modules, including cross-attention, CBAM, SE block, Mish activation, and Group Normalization.

**Table 18:** Ablation study results of the proposed CoAT-AgriLite model.

Model variant	Acc (%)	Pr (%)	Recall (%)	F1 score (%)
Model A (Full)	99.58	99.41	99.18	99.29
Model B (Cross-Attn)	98.94	98.7	98.55	98.62
Model C (CBAM)	99.06	98.8	98.7	98.75
Model D (SE)	99.14	98.91	98.77	98.84
Model E (ReLU → Mish)	99.2	99	98.83	98.91
Model F (–GroupNorm)	99.02	98.72	98.6	98.66

As for the comparison between the cross-attention-based model (Model A) and the one without cross-attention (Model B), the most significant degradation of performance in all metrics (accuracy: 98.94%; F1-score: 98.62%) on the test set highlighted the importance of the mechanism in integrating local and global features to discriminate disease accurately. Moreover, the removal of channel and spatial attention modules, for example, CBAM (Model C) and SE block (Model D), led to significant performance decreases in precision, recall, and F1-score, indicating their effectiveness in improving feature representation and focusing on the disease-specific region by the model. Replacing Mish with the conventional ReLU activation (Model E) yields a slight performance decrease, suggesting that Mish’s non-linearity helps better learning dynamics for subtle disease cues. Dropping Group Normalization (Model F) also resulted in slight drops in all performance metrics, demonstrating its stabilizing role, especially under varied training settings. In spite of these advancements, the ablation study in Table 5 also shows that the feasibility is not sufficient. It increases the overall model complexity, the number of parameters, and the FLOPs by introducing multi-attention and normalization mechanisms, which may create deployment issues for the application in real-time or edge practical scenarios with limited computation. In addition, the increase in performance achieved by individual modules, albeit statistically significant, can sometimes be relatively small compared to the extra computational cost introduced. This trade-off suggests the need for future work to explore model compression, pruning, or lightweight alternatives in exchange for accuracy and efficiency in practical, field-ready applications.

**Table 19:** Performance comparison of standalone and hybrid models.

Model type	Model	Acc (%)	DSC (%)
CNN-only	RegNetY-B3-GE	96.43	96.31
CNN-only	DarkNet53-SCSE	96.89	96.91
Transformer-only	BEiT	97.38	97.56
Transformer-only	PVTv2	97.81	97.83
Hybrid (proposed)	CoAT-AgriLite	99.37	99.37

The proposed CoAT-AgriLite model was explicitly compared with standalone CNN-based and Transformer-based architectures to quantify the contribution of hybridization, as summarized in Table 19. RegNetY-B3-GE and DarkNet53-SCSE were selected as representative convolution-only models, while BEiT and PVTv2 served as transformer-only baselines. Experimental results from testing and cross-validation indicate that CNN-only architectures achieved testing accuracies in the range of 96.43%–96.89%, demonstrating strong local feature extraction capability but limited global contextual modelling. Transformer-only models exhibited improved performance (97.38%–97.81%) due to their ability to capture long-range dependencies; however, the absence of strong convolutional inductive biases constrained their effectiveness for fine-grained disease characterization. In contrast, the proposed hybrid CoAT-AgriLite model achieved a testing accuracy and DSC of 99.37%, reflecting the complementary benefits of combining convolutional priors with attention-based global modelling. Although the Dice Similarity Coefficient (DSC) is traditionally employed in segmentation tasks, it is mathematically equivalent to the F1-score when derived from classification confusion matrices. Accordingly, DSC values are reported here as macro-averaged F1-scores to enable fair comparison with prior studies. As shown in Table 14, CoAT-AgriLite substantially outperforms both CNN-based (DSC: 96.31%–96.91%) and Transformer-based (DSC: 97.56%–97.83%) models, thereby confirming the effectiveness of the proposed hybrid architecture.

## Discussion

The proposed model CoAT-AgriLite has a superior performance due to its feature-level hybrid architecture that combines fine-grained lesion characteristics and long-range context dependencies. It is global reasoning which is crucial in fruit disease classification as the disease pattern can have similarities and thus needs to be understood at an image level rather than a local level. The combination of convolutional inductive priors and attention-based modelling allows for accurate discrimination of subtle and diffuse disease presentations. As opposed to using only CNN methods, the proposed framework is able to capture global contextual information more effectively. On the other hand, when we train transformer-only models on moderate-sized datasets, they have weak lesion localization, which causes overfitting. CoAT-AgriLite alleviates these concerns by placing convolutional priors directly in the attention mechanism, which yields consistent and meaningful gains over existing architectures. The proposed framework’s reliability is further supported by the analysis of Grad-CAM, which identifies lesions, discolorations and other disease-relevant areas that strongly influence the model’s predictions. We can conclude that the classification decisions are not due to spurious background artifacts. Gaining user trust through such explainability enhances the potential for practical use of automated disease diagnosis systems in agricultural settings.

**Table 20:** Classification accuracy comparison of proposed and other SOTA models.

Authors	Model	Classification Acc (%)
^18^	DeepLabV3	93.21
^19^	HCA-YOLOv8	98.12
^21^	YOLOv5s	95.6
^23^	DenseNet-121	98.56
^24^	DenseNet-201 + AlexNet	99.2
^25^	DenseNet-121/InceptionV3	99.12
^26^	GoogLeNet + VGG16	99.21
^28^	MSMP-CNN	98.5
^33^	Hybrid OACapsNet	99.19
^34^	DenseNet121 + SE and YOLOv8n	98.25
Proposed	CoAT-AgriLite	99.37

As can be observed from Table 20, CoAT-AgriLite outperforms all existing state-of-the-art techniques in classification accuracy and thus achieved the good accuracy among all of them. Note that the existing models are either CNN-based or Transformer-based or Hybrid. The ensemble and multi-scale models use different backbone networks to extract complementary features, while the proposed method achieves better performance by integrating local texture modelling and global contextual reasoning in a unified and lightweight framework. The ability of a carefully designed hybrid structure to outperform heavier ensemble structures is repeated. CoAT-AgriLite is feasible for real-world agricultural disease diagnosis due to balance between accuracy, computational efficiency and interpretability. The present evaluation is however performed on public datasets that are collected from controlled or semi-controlled conditions and performance in open-field environments with cluttered backgrounds and illumination variation and sensor noise may differ. While the CoAT-AgriLite has shown good results on the Kaggle fruit disease dataset, we must remember that public datasets are usually collected from controlled setups or semi-controlled setups. The image data collection from controlled and semi-controlled setups takes place in a scenario with a clean background and less variability in the environmental conditions and COVID-19. Moreover, the fuzzy background with images of fruits is highly unrealistic in the agricultural environment or natural environment under open-field conditions, and a preferable environment for robust performance investigation and assessment would be the open-field where images are affected by cluttered backgrounds, illumination variations, occlusion, and sensor noise. Furthermore, the outcomes of this work can be seen as an upper bound to the findings in an open-field scenario deployment or in a difficult robust realtime environment deployment. In our future works we will validate on the collected challenging datasets.

It is essential to address that fruits are fundamentally three-dimensional objects, however the present study works on single-view two-dimensional images. In this regard, the model choice is reliable with the acquisition techniques of broadly used public datasets and practical agricultural inspection scenarios, such as mobile-phone-based diagnosis and automated sorting systems, where only one visible surface of the fruit is typically captured. Therefore, the proposed study is used to address the disease symptoms existing on the experiential surface rather than infer the complete three-dimensional health status of the fruit. While localized disease manifestation on a single side may not always represent the overall condition of the fruit, however the incorporation of attention mechanisms and XAI ensures that the predictions are directed by visible lesion features. The multi-view and 3D-aware disease imaging are acknowledged as important extensions and those are considered as promising directions for future endeavour.

## Conclusion

The primary objective of this study was to develop an explainable and accurate DL model for the multi-class classification of fruit diseases. For this purpose, we conducted extensive experiments with a range of pre-trained models such as RegNetY-B3-GE, DarkNet53-SCSE, BEiT, and PVTv2, and compared their advantages and disadvantages in detecting disease features from fruit images. Based on this analysis, we presented the CoAT-AgriLite model, a new hybrid architecture that combines Convolutional layers for local feature extraction and transformer-based attention mechanisms for capturing global contextual relationships. One significant new contribution of our work is the seamless integration of cross-scale and orientation-aware attention in a lightweight architecture, enabling the model to handle the complex and diverse appearances of fruit diseases effectively in practical situations. We also used another explainable AI method, Grad-CAM, to give visual explanations of the model predictions to add more transparency for users. Robust experimental results proved the efficacy of the method. The classification accuracy obtained was 99.37% which is better than all the pretrained ones. The Indian Institute of Science (IISc) researchers successfully designed a robust thermalization protocol to protect quantum states from environmental noise. This device-independent approach proposes a device with which one can transfer the quantum states accurately. The convolutional and transformer-based modules leveraged local and global characteristics. Hence, the model remained good for various fruit diseases that expressed complex and diverse visual appearances in the environment. Additionally, Grad-CAM-based explainable AI modules assuage the existing worry concerning transparency and interpretability of automated disease detection, as it allows us to see into the workings of the model.

Nonetheless, limitations of this proposed study are quite obvious. This limitation could be mainly because of regime-specific reliance on a single imaging modality and a manual selection of dataset with which we were not necessarily able to capture all available environmental, lighting variations and types of rare diseases from various agricultural regions. When deployed in new or unseen real-world settings, the generalization and robustness of the model may be affected. Further, the current model has only been tested on static images, which fail to represent the dynamics and temporal aspects of real disease progression. A current limitation of the proposed approach is its reliance on single-view images, which may not fully capture disease patterns distributed across the entire three-dimensional surface of fruits; future work will investigate multi-view fusion and 3D-aware imaging to overcome this limitation. To fix these issues, the future work focuses on a few things. Initially, our plan will be to augment the available dataset with the incorporation of several additional samples that were collected from other geographical regions and under several environmental and lighting conditions. We will also be using samples that represent rarer disease classes and newer ones that are emerging. Our intentions are also to take multi-modality into account. For example, hyperspectral, thermal or fluorescence imaging, which may provide richer and complementary information to detect disease. It is important to understand domain adaptation and transfer learning to ensure the model maintains high performance when applied to unseen data.

## Data Availability

The datasets used during the current study are available from the corresponding author on reasonable request.
